# 4‐phenylbutyric acid promotes hepatocellular carcinoma via initiating cancer stem cells through activation of PPAR‐α

**DOI:** 10.1002/ctm2.379

**Published:** 2021-05-01

**Authors:** Shu‐Zhen Chen, Yan Ling, Le‐Xing Yu, Yu‐Ting Song, Xiao‐Fei Chen, Qi‐Qi Cao, Han Yu, Can Chen, Jiao‐Jiao Tang, Zhe‐Cai Fan, Yu‐Shan Miao, Ya‐Ping Dong, Jun‐Yan Tao, Satdarshan P.S. Monga, Wen Wen, Hong‐Yang Wang

**Affiliations:** ^1^ National Center for Liver Cancer Second Military Medical University Shanghai China; ^2^ International Cooperation Laboratory on Signal Transduction Eastern Hepatobiliary Surgery Hospital Second Military Medical University Shanghai China; ^3^ Model Animal Research Center Nanjing University Nanjing Jiangsu Province China; ^4^ School of Pharmacy Second Military Medical University Shanghai China; ^5^ State Key Laboratory of Oncogenes and Related Genes Shanghai Cancer Institute Renji Hospital School of Medicine Shanghai Jiaotong University Shanghai China; ^6^ Fujian Medical University Fuzhou Fujian Province China; ^7^ Cancer Research Center The First Affiliated Hospital of University of Science and Technology of China Hefei Anhui Province China; ^8^ Department of Pathology University of Pittsburgh Pittsburgh Pennsylvania USA; ^9^ Department of Medicine University of Pittsburgh Pittsburgh Pennsylvania USA

**Keywords:** 4‐phenylbutyric acid (4‐PBA), cancer stem cell (CSC), hepatocellular carcinoma (HCC), peroxisome proliferator‐activated receptor‐α (PPAR‐α)

## Abstract

**Background and aims:**

4‐phenylbutyric acid (4‐PBA) is a low molecular weight fatty acid that is used in clinical practice to treat inherited urea cycle disorders. In previous reports, it acted as a chemical chaperone inhibiting endoplasmic reticulum (ER) stress and unfolded protein response signaling. A few studies have suggested its function against hepatic fibrosis in mice models. However, its role in hepatocarcinogenesis remained unknown.

**Methods:**

4‐PBA was administered alone or in combination with diethylnitrosamine to investigate its long‐term effect on liver tumorigenesis. The role of 4‐PBA in oncogene‐induced hepatocellular carcinoma (HCC) mice model using sleeping beauty system co‐expressed with hMet and β‐catenin point mutation (S45Y) was also observed. RNA‐seq and PCR array were used to screen the pathways and genes involved. *In vitro* and *in vivo* studies were conducted to explore the effect of 4‐PBA on liver and validate the underlying mechanism.

**Results:**

4‐PBA alone didn't cause liver tumor in long term. However, it promoted liver tumorigenesis in HCC mice models via initiation of liver cancer stem cells (LCSCs) through Wnt5b‐Fzd5 mediating β‐catenin signaling. Peroxisome proliferator‐activated receptors (PPAR)‐α induced by 4‐PBA was responsible for the activation of β‐catenin signaling. Thus, intervention of PPAR‐α reversed 4‐PBA‐induced initiation of LCSCs and HCC development *in vivo*. Further study revealed that 4‐PBA could not only upregulate the expression of PPAR‐α transcriptionally but also enhance its stabilization via protecting it from proteolysis. Moreover, high PPAR‐α expression predicted poor prognosis in HCC patients.

**Conclusions:**

4‐PBA could upregulate PPAR‐α to initiate LCSCs by activating β‐catenin signaling pathway, promoting HCC at early stage. Therefore, more discretion should be taken to monitor the potential tumor‐promoting effect of 4‐PBA under HCC‐inducing environment.

## Graphical abstract

1. 4‐PBA could promote HCC at early stage of tumor development *in vivo*.
2. 4‐PBA initiated CSCs via activating Wnt5b‐Fzd5‐β‐catenin signaling pathway dependent of PPAR‐α activation.
3. 4‐PBA could not only regulate the expression of PPAR‐α transcriptionally, but also stabilize it via direct binding.

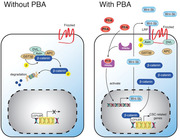

## 1 INTRODUCTION

Liver cancer is a common solid tumor worldwide with nearly 800,000 new cases annually and ranks as the third leading cause of cancer‐related deaths.^1^, ^2^ Hepatocellular carcinoma (HCC) alone accounts for 90% of all primary liver cancer cases with poor prognosis. Identification of chemical compounds that pose great threat to HCC is an effective approach for HCC prevention.^3^ During the past years, various risk factors of HCC were identified, including ingestion of the fungal metabolite Aflatoxin B1, hepatitis B virus infection, hepatitis C virus infection, nonalcoholic fatty liver disease (NAFLD), and alcohol intake.^4^, ^5^, ^6^, ^7^, ^8^ Hepatocytes injury caused by these risk factors initiated the damage repair mechanism and created a chronic inflammatory environment which facilitated the initiation of liver cancer stem cells (LCSCs) and the subsequent budding of liver cancer.^9^, ^10^

4‐phenylbutyric acid (4‐PBA) is a small molecular weight fatty acid approved by the Food and Drug Administration (FDA) as a well‐tolerated drug for patients with urea cycle disorders and hyperammonemia (450 mg/kg/day in patients weighing less than 20 kg, or 9.9–13.0 g/m^2^/day in larger patients), where it plays the role of an ammonia scavenger.^11^, ^12^, ^13^ It has been regarded as a novel chemical chaperone to reduce endoplasmic reticulum (ER) stress due to its role in stabilizing protein conformation, improving ER folding capacity, and facilitating the trafficking of proteins.^14^, ^15^ With the recognition of ER stress dysregulation caused human diseases, the role of 4‐PBA in obesity,^16^ diabetes,^14^ ischemic injury,^17^ and liver fibrosis^18^ *etc*. has been explored, providing novel drug targets and promising strategies for therapeutic intervention.^19^, ^20^

4‐PBA was identified as a suppressor of tumor cell growth decade ago.^21^, ^22^ However, a recent study has found that 4‐PBA promoted gastric cancer cell migration via mediating HER3/HER4 upregulation. Since 4‐PBA is derived from butyric acid, which is produced in the digestive tract by colonic bacteria,^23^ it could be involved in liver diseases where gut‐derived nutrients and microbiota‐derived signals constantly flood via the portal system.^24^, ^25^, ^26^ Several studies have indicated a protective role of 4‐PBA in liver injury. Data showed that 4‐PBA prevented CCl_4_‐induced hepatic fibrosis via impeding hepatic inflammatory response and hepatic stellate cells activation.^18^ It could attenuate ER stress‐mediated apoptosis and protect hepatocytes from intermittent hypoxia‐induced injury.^27^ Meanwhile, it was also responsible for reducing hepatocellular lipid accumulation and lipotoxicity through induction of autophagy.^28^ However, the above studies only focused on short‐term effect of 4‐PBA on liver injury. The long‐term effect of 4‐PBA on liver tumorigenesis remained unknown.

In this study, we evaluated the role of 4‐PBA in HCC carcinogenesis. Our data showed that 4‐PBA significantly increased liver tumor burden in diethylnitrosamine (DEN) or 3,5‐diethoxycarbonyl‐1,4‐dihydrocollidine (DDC) supplemented HCC models when administered at an early stage, even though liver inflammation and fibrosis seemed alleviated. 4‐PBA functioned via initiation of LCSCs by activating Wnt5b‐Fzd5 mediating β‐catenin pathway in a peroxisome proliferator‐activated receptors (PPAR)‐α dependent manner. Further study revealed that 4‐PBA not only upregulated the expression of PPAR‐α, but also stabilized it via directly binding to it. Moreover, intervention of PPAR‐α with inhibitor or short hairpin RNA (shRNA) reversed the tumor‐promoting effect of 4‐PBA. Meanwhile, high PPAR‐α expression predicted poor prognosis in HCC patients. Our study suggested that 4‐PBA could cause liver tumorigenesis in HCC‐inducing environment via initiating LCSCs. Therefore, more caution should be taken for long‐term use of 4‐PBA in clinical practice.

## 2 MATERIALS AND METHODS

### 2.1 Experimental animal models

Male *C57BL/6* and nude mice were purchased from Shanghai Experimental Center of Chinese Academy of Science and maintained under pathogen‐free conditions. Each group includes more than six mice except otherwise indicated. The HCC model in mice was induced by injection of DEN (Sigma‐Aldrich, St. Louis, MO, 25 mg/kg) intraperitoneally at age of 15 days. A single injection of DEN was followed by administration of carbon tetrachloride (CCl_4_) 1 ml/kg per week. Mice were fed without or with 1 g/kg/day of 4‐PBA supplemented in the drinking water. Randomly grouped mice were infected with adeno‐associated virus (AAV)‐shNC (3  ×  10^11^ IU/animal; *i.p*., *n*  =  5) or AAV‐shPpara (3  ×  10^11^ IU/animal; *i.p*., *n*  =  6). AAV was constructed and purchased from Obio technology company (Shanghai, China).

For hMetS45Y‐β‐catenin and hMet model, hydrodynamic tail vein injections were performed. Briefly, 20 mg pT3‐EF5a‐hMet‐V5 or pT3‐EF5a S45Y‐β‐catenin‐Myc or the combination of pT3‐EF5a‐hMet‐V5 and pT3‐EF5a‐S45Y‐β‐catenin‐Myc, accompanied with the sleeping beauty (SB) transposase were diluted in 2 ml of normal saline (0.9% NaCl) in a ratio of 25:1 and injected into the tail vein of 6 to 8‐week‐old *C57BL/6* mice in 5–7 seconds.

All animals received humane care according to the criteria outlined in the "Guide for the Care and Use of Laboratory Animals," and the animal experiments protocols were approved by the Institutional Animal Care and Use Committee of Second Military Medical University.

### 2.2 Drug affinity responsive target stability assay

Studies were performed as previously reported.^29^ Cells were lysed and treated with various concentrations of 4‐PBA followed by digestion with pronase and stopped by SDS‐PAGE loading buffer. Samples were run on a SDS‐PAGE electrophoresis gel for silver staining. 4‐PBA‐protected candidate proteins were identified and confirmed by western blotting. The details are described in supplementary materials.

### 2.3 Statistical analysis

All data are presented as means with the standard deviation unless otherwise indicated. For comparisons of two groups, two‐tailed unpaired Student's *t*‐test was performed. Cumulative survival time was calculated with the Kaplan‐Meier method and analyzed by the log‐rank test. Univariate and multivariate analyses were based on the Cox proportional hazards regression model. A *p*‐value < 0.05 was considered statistically significant. Statistical calculations were performed using SPSS 16.0 software (SPSS, Chicago, IL, USA).

## 3 RESULTS

### 3.1 4‐PBA promoted chemical carcinogen‐induced and driver gene‐induced HCC carcinogenesis *in vivo*

**FIGURE 1:**
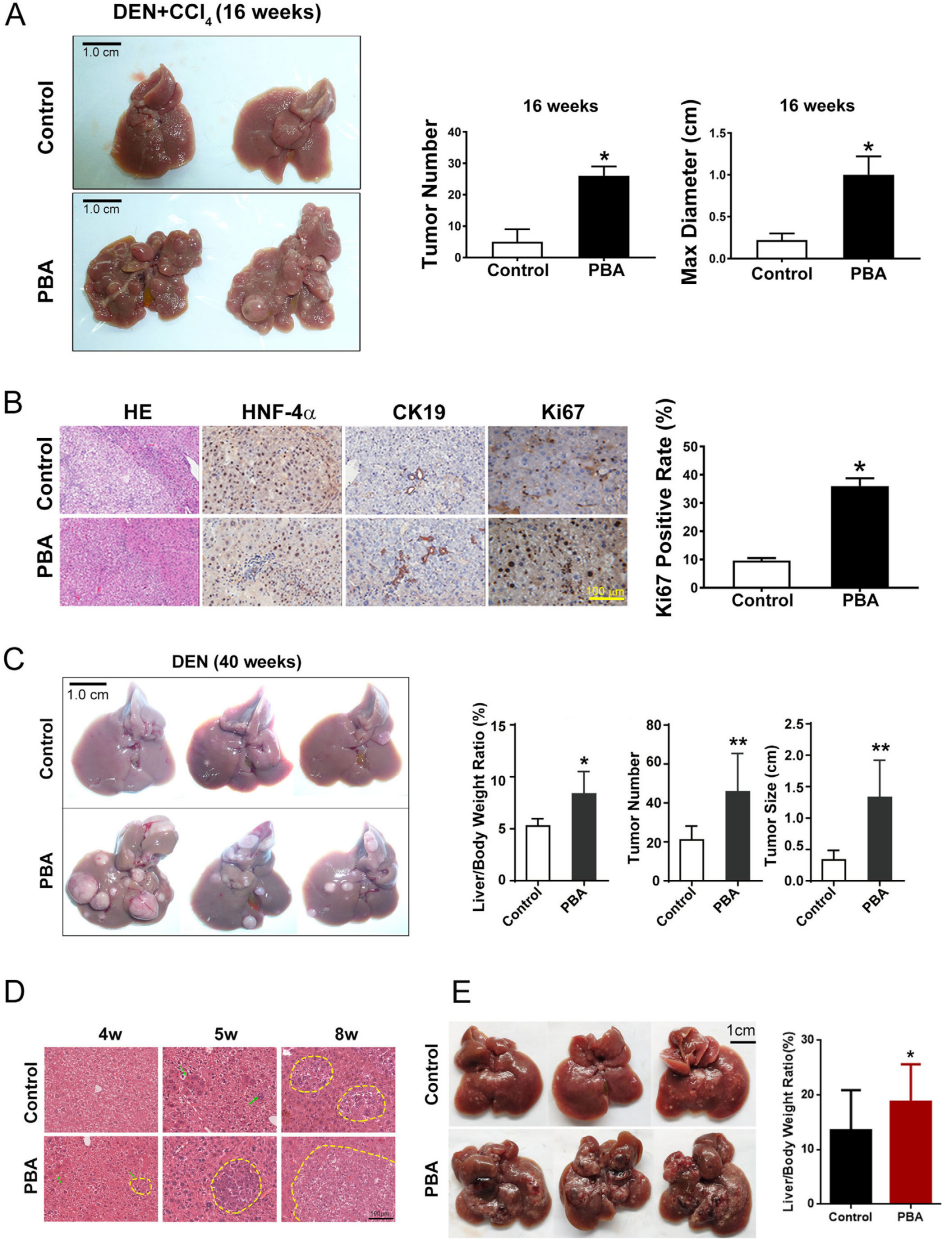
4‐PBA promoted chemical carcinogen‐induced and driver gene‐induced HCC in mice model. (A) Representative images of the liver in DEN plus CCl_4_ model after 16 weeks. Scale bar: 1 cm. Tumor number and maximum diameter of tumors in PBA‐treated group and the control group were measured. *p < 0.05. (B) H&E staining, HNF4α, CK19, and Ki67 staining of representative liver sections from both groups in DEN plus CCl_4_ model. Scale bar: 100 μm. Ki67 positive staining rates were calculated. *p < 0.05. (C) Representative images of livers in DEN induced HCC model after 40 weeks in PBA‐treated group and the control group. Scale bar: 1 cm. Liver/body weight ratio, tumor number, and tumor size were measured. *p < 0.05, **p < 0.01 (D) H&E staining of representative liver section in hMetS45Y‐β‐catenin model at 4 weeks, 5 weeks, and 8 weeks, respectively. Yellow circle displays microscopic foci of basophilic hepatocytes at 4 weeks and 5 weeks and tumor nodules at 8 weeks. Scale bar: 100 μm. (E) Representative images of liver in hMetS45Y‐β‐catenin model at 8 weeks in PBA treated group and the control group. Scale bar: 1 cm. Liver/body weight ratio in both groups was measured. *p < 0.05

To test the effect of 4‐PBA on HCC development *in vivo*, male *C57BL/6* mice were fed with a normal diet for 4 months supplemented with or without 4‐PBA (1.0 g/kg/day) in the drinking water. No liver tumor was observed with 4‐PBA treatment alone after 40 weeks (Figure S1A), indicating that 4‐PBA was incapable of initiating HCC. We further used chemical‐induced HCC model via a single injection of DEN followed by repeated administration of carbon tetrachloride (CCl_4_). A dramatic potentiation of liver tumor incidence indicated by more and larger tumors was observed in 4‐PBA supplemented mice when evaluated at the time point of 16 and 20 weeks (Figures 1A, S1B and S1C). Hepatic nuclear factor 4 alpha (Hnf‐4α), a marker of differentiated hepatocyte, was down‐regulated in 4‐PBA treated liver, whereas more ductular reaction marked by CK19 and enhanced cell proliferation marked by Ki‐67 were observed at 16 weeks (Figure 1B).

As liver carcinogenesis induced by DEN alone in mice closely mimics the pathology during human liver cancer initiation and progression, we observed the effect of 4‐PBA in a single dose DEN treated mouse model. Consistent with the previous model, tumor incidence was also enhanced in 4‐PBA supplemented mice, suggested by enlarged liver/body weight ratio, tumor number, and tumor size (Figure 1C). DDC could induce severe cholestatic liver injury with strong ductular reaction; however, DDC alone rarely initiated liver tumorigenesis. When mice fed with DDC diet were sacrificed after 57 weeks, 4‐PBA treated mice have developed visible liver tumors, while no tumor was observed in the control group (Figure S1D). The above models demonstrated that 4‐PBA promoted chemical carcinogen‐induced HCC.

We next introduced concomitantly expressed hMet and an S45 to tyrosine (Y) mutant form of β‐catenin (S45Y‐β‐catenin, with a Myc tag) using SB transposon/transposase (henceforth referred to as hMetS45Y‐β‐catenin model) to *C57BL/6* mice via hydrodynamic tail vein injection to model human HCC in mice as previously described.^30^ In this model, clusters of small microscopic foci with basophilic hepatocytes could be detected as early as 4 weeks after 4‐PBA treatment (Figure 1D). Myc‐tag could be detected in both groups at this time (Figure S1E). Notable tumor burden with areas of necrosis could be observed in 4‐PBA treated group 8 weeks after plasmids injection, demonstrated by larger macroscopic tumors and liver/ body weight ratio (Figure 1E). The above data indicated that 4‐PBA could also accelerate tumorigenesis in short‐term HCC model. Taken together, 4‐PBA promotes liver tumor development both in long‐term chemical carcinogen‐induced HCC models and short‐term driver gene‐induced model.

### 3.2 4‐PBA gave rise of HCC at early stage of tumor development

**FIGURE 2:**
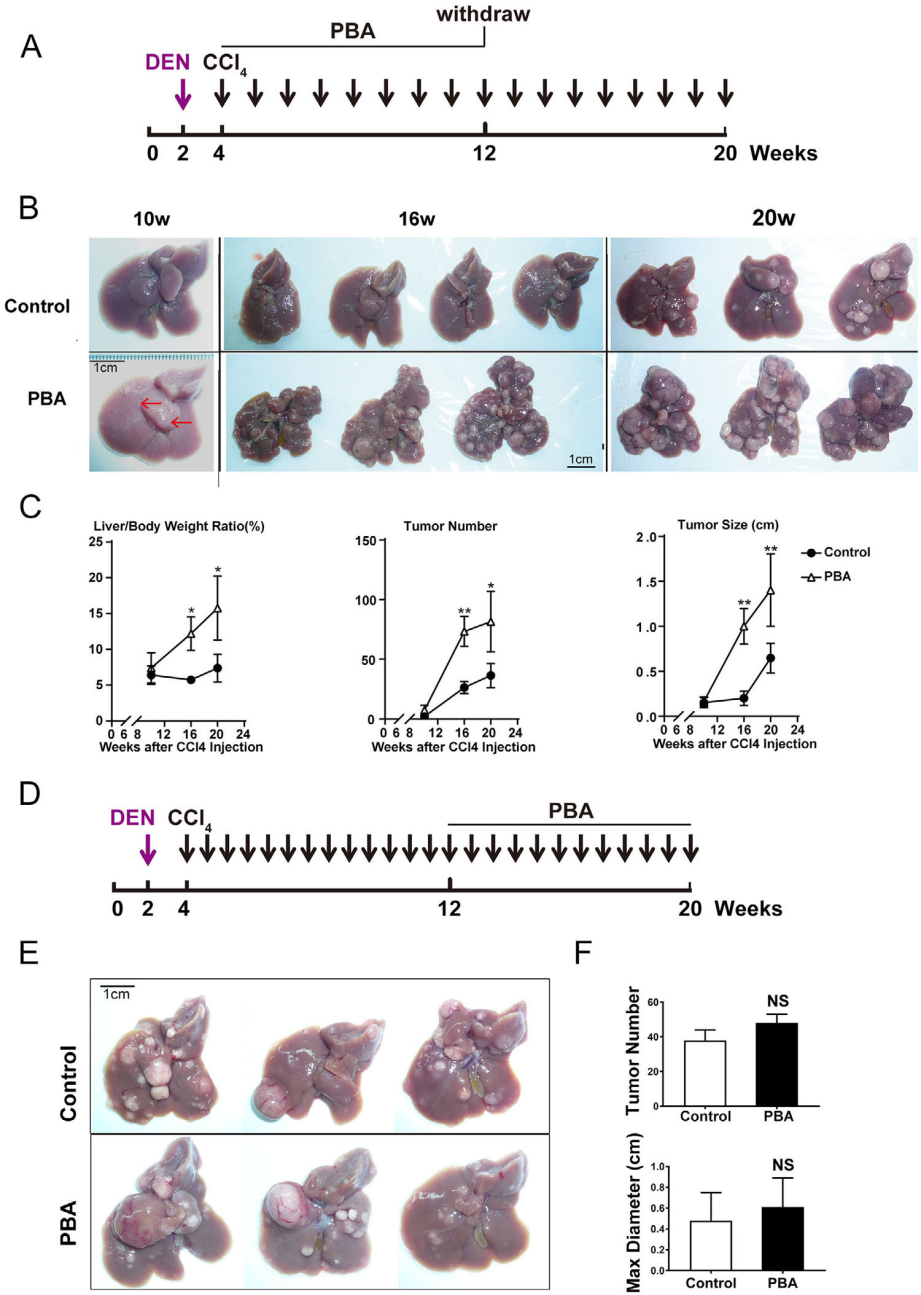
4‐PBA gave rise of HCC at early stage of tumor development. (A) Schematic experimental outline of 4‐PBA administration at early stage of tumor development. (B) Representative images of livers at 10 weeks, 16 weeks, and 20 weeks as indicated in 2A. Scale bar: 1 cm. (C) Liver/body weight ratio, tumor number, and tumor size were measured in PBA‐treated group and the control group at 16^th^ week. *p < 0.05, **p < 0.01. (D) Schematic experimental outline of 4‐PBA administration at later stage of tumor development. (E) Representative images of livers at 20 weeks as indicated in 2D. Scale bar: 1 cm. (F) Tumor number and maximum diameter of tumors in 2E were measured in PBA‐treated group and the control group.

To define the time frame when 4‐PBA affected liver tumor development, we altered the time of 4‐PBA administration in DEN‐induced HCC model (Figures 2A and 2D). Liver tumorigenesis was markedly increased when 4‐PBA was supplemented 2 weeks after DEN injection (early stage of tumor development), even though it was withdrawn 8 weeks later (Figure 2A). In 4‐PBA group, macroscopic tumors occurred at as early as the 10^th^ week, and abundant tumors could be observed at 16^th^ week (Figure 2B). Moreover, liver/body weight ratio, tumor number, and tumor size were significantly increased compared with control group (Figure 2C). However, when 4‐PBA was supplemented at later stage after DEN injection for 10 weeks (later stage of tumor development), liver tumorigenesis in 4‐PBA combined group was not significantly affected, suggested by comparable tumor number and maximum tumor size with control group (Figures 2D‐2F). These two models suggested a synergic effect of 4‐PBA with DEN in early stage of liver cancer formation.

### 3.3 4‐PBA promoted tumorigenesis via initiation of cancer stem cells instead of inflammation and ER stress‐dependent way

We tested the effect of 4‐PBA *in vitro* and found that 4‐PBA does not significantly accelerate the proliferation of hepatocyte cell line L02 and HCC cell line HepG2 (Figures S2A and S2B). Established data showed that the inflammatory microenvironment played an important role in liver cancer initiation.^31^, ^32^ Previous study has revealed a protective role of 4‐PBA in hepatic fibrosis via inhibiting hepatic inflammatory responses.^18^ In our study, liver fibrosis caused by CCl_4_ was ameliorated by 4‐PBA administration measured by Sirius red and α‐SMA expression detection (Figures S2C and S2D). IHC staining of F4/80 revealed rare increase of Kupffer cells in the liver of 4‐PBA supplemented mice (Figure S2E). Meanwhile, similar levels of TNF‐α were presented in both groups (Figure S2F). In 4‐PBA alone treated mice, alanine aminotransferase (ALT) and aspartate aminotransferase (AST) level were not significantly altered (Figure S2G). Whereas, they were significantly lower in 4‐PBA supplemented mice in DEN plus CCl_4_ model (Figure S2H), suggesting a possibly relieved inflammation environment. Next, we determined the role of 4‐PBA on ER stress and apoptosis in the above model. 4‐PBA reduced p‐PERK at 2 weeks after treatment, but it was restored 4 weeks later (Figure S2I). TUNEL assay revealed that DEN‐induced apoptosis was significantly reduced in the liver of 4‐PBA supplemented mice (Figure S2J). However, the expression of CHOP, a downstream molecule of unfolded protein response triggering apoptosis, was not inhibited by 4‐PBA (Figure S2I). These data indicated that 4‐PBA promoted liver tumorigenesis via mechanism other than inflammation and ER stress‐dependent pathways.

**FIGURE 3:**
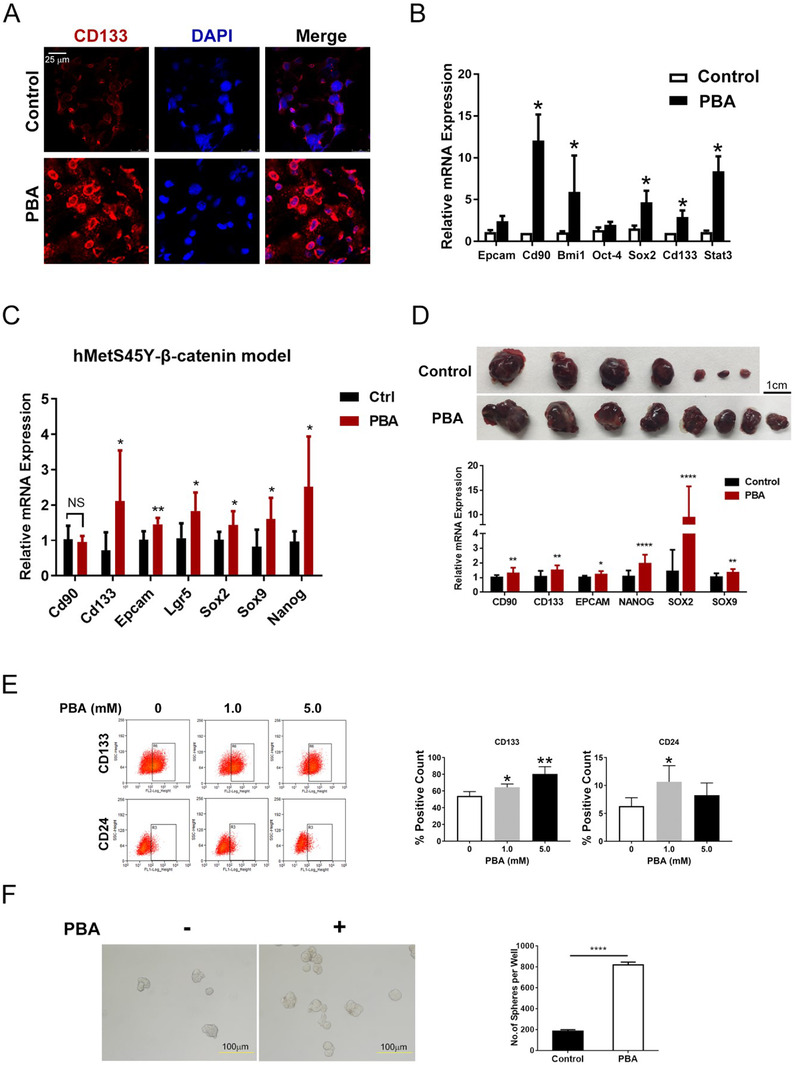
4‐PBA promoted tumorigenesis via initiation of cancer stem cells. (A) Representative photos of CD133 immunofluorescence of the fresh liver section in DEN plus CCl_4_ HCC model after 6 weeks with or without PBA. Scale bar: 25 μm. (B) Relative expression of *Epcam*, *Cd90*, *Bmi1*, *Oct‐4*, *Sox2*, *Cd133*, and *Stat3* in DEN plus CCl_4_ mice liver after 6 weeks. *p < 0.05. (C) Relative expression of *Cd90*, *Cd133*, *Epcam*, *Lgr5*, *Sox2*, *Sox9*, and *Nanog* in hMetS45Y‐β‐catenin model after 8 weeks. *p < 0.05, **p < 0.01. (D) Representative pictures of tumors in nude mice derived from Huh7 (2 × 10^6^ cells) 14 days after cell implantation treated with or without PBA treatment. Scale bar: 1 cm. Relative expression of *CD90*, *CD133*, *EPCAM*, *NANOG*, *SOX2*, and *SOX9* in tumors of nude mice. *p < 0.05, **p < 0.01, ****p < 0.0001. (E) The change of CD133 or CD24 positive Huh7 cells after different doses of PBA treatment for 24 h is detected by flow cytometry analysis. The bar chart shows the quantification of CD133 and CD24 positive cells. *p < 0.05, **p < 0.01. (F) Representative photos of spheres formed with Huh7 cells treated with or without PBA for 5 days. The bar chart shows the average amount of spheres formed per well. ****p < 0.0001

Cells with stem‐cell properties are involved in HCC initiation.^33^ In human, hepatic stem/progenitor cells are markedly elevated in chronic liver diseases.^34^ Impairment in hepatocyte proliferation may cause the expansion of stem/progenitor cells called “ductular reactions.”^35^ In DEN‐induced HCC model, higher expansion of CK19 and SOX9 positive cells was detected as early as 2 weeks after 4‐PBA treatment (Figure S3A). Meanwhile, relative mRNA of α‐Fetoprotein （Afp） and long noncoding RNA‐H19 (H19), both imprinted oncofetal gene, were significantly upregulated in the liver of 4‐PBA treated group (Figure S3B). Higher CD133 expression was detected in fresh frozen sections from the liver of 4‐PBA supplemented mice for 6 weeks (Figure 3A), along with the elevation of other CSC‐related genes, including *Epcam*, *Cd90*, *Bmi‐1*, *Oct‐4*, *Sox‐2*, *Cd133*, and *Stat3* (Figure 3B). In DDC‐diet model, stronger ductular reaction indicated by SOX9 and CK19 staining could also be observed at 4 weeks in 4‐PBA supplemented mice (Figure S3C), with elevated expression of EpCAM in liver sections (Figure S3D). These data suggested the activation of LCSCs under the effect of 4‐PBA in chemical‐induced HCC model. In hMetS45Y‐β‐catenin model, six of seven CSC markers were upregulated in 4‐PBA‐treated mouse liver after 4 weeks (Figure 3C). More Cd133 and Epcam positive cells could be observed in these liver tissues (Figures S3E and S3F). In nude mice xenograft model, HCC cell line Huh7 was injected subcutaneously, and 4‐PBA was supplemented in drinking water the day after. As expected, 4‐PBA played tumor‐promoting role with more and larger tumors formed than the control group (Figures 3D and S3G). Meanwhile, a subset of stem‐cell‐related genes such as *Cd90* and *Cd133* were significantly upregulated in tumor tissues of 4‐PBA‐treated group (Figure 3D). Furthermore, limiting dilution assay was performed with HepG2, which also exhibited enhanced tumor initiating capacity with 4‐PBA treatment (Figure S3H).

We also demonstrated the role of 4‐PBA on CSCs *in vitro*. Flow cytometry analysis detected an increase of CD133 and CD24 positive cells in Huh7 cell line cultured with 4‐PBA (Figure 3E). In sphere formation assay, more spheres were formed when 4‐PBA was supplemented in culture medium (Figure 3F). Similarly, 4‐PBA induced more colonies of HepG2 in soft agar (Figure S3I). Therefore, we concluded that 4‐PBA played a tumor‐promoting role in liver via initiating LCSCs other than inflammation and ER stress‐dependent way.

### 3.4 4‐PBA regulated CSCs via Wnt5b‐Fzd5 mediating β‐catenin signaling pathway

**FIGURE 4:**
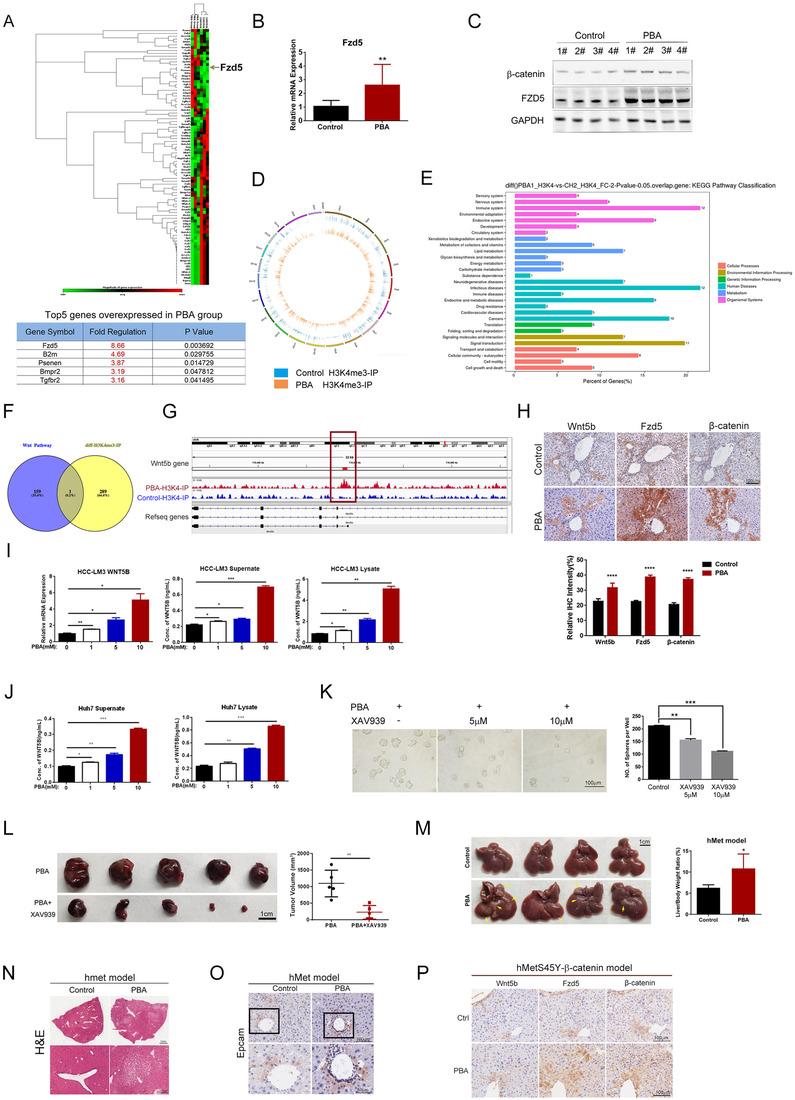
4‐PBA regulated CSCs via activating Wnt5b‐Fzd5 mediating β‐catenin signaling pathway. (A) Heatmap of mouse stem cell signaling‐related genes determined by PCR array. Each group includes three samples. The arrow points at the expression of Fzd5, which is significantly increased in 4‐PBA treated group. The table represented top five overexpressed stem cell‐related genes in liver samples of DEN‐induced HCC model with 4‐PBA treated for 6 weeks compared with the control group. (B) Relative expression of *Fzd5* in liver samples of DEN model with 4‐PBA supplemented for 6 weeks was determined by RT‐PCR. **p < 0.01. (C) The expression of β‐catenin and Fzd5 was detected by western blot. (D) CHIP‐seq was conducted in primarily separated hepatocyte from mice in DEN model treated with or without 4‐PBA for 6 weeks. H3K4me3 antibody was used as the binding protein. Gene distribution heat map of CHIP‐seq was presented. (E) KEGG pathway classification of the differential genes in CHIP‐seq was presented. (F) Wnt5b is the only overlapped gene of wnt pathway among varied genes detected in CHIP‐seq. (G) *Wnt5b* was detected as the differential gene among Wnt signaling‐related genes as the red square indicated. (H) Top: The expression of Wnt5b, Fzd5, and β‐catenin in serial section of liver in DEN model was detected by IHC. Scale bar: 100 μm. Down: The relative IHC intensity of Wnt5b, Fzd5, and β‐catenin. (I) MHCC‐LM3 was cultured in serum‐free medium supplemented with different doses of 4‐PBA (0, 1, 5, 10 mM) for 24 h. mRNA level of Wnt5b is determined by RT‐PCR. *p < 0.05, **p < 0.01. Wnt5b concentration in supernatant and cell lysate was measured by ELISA. (J) Huh7 was cultured in serum‐free medium supplemented with different doses of 4‐PBA (0, 1, 5, 10 mM) for 24 h. Wnt5b concentration in supernatant and cell lysate was measured by ELISA. (K) Huh7 was cultured in medium with PBA and different doses of XAV939 (0, 5, 10 μM) for 7 days. Representative photographs and the number of spheres formed in each group were presented. Scale bar: 200 μm. (L) Representative pictures of tumors in nude mice derived from Huh7 cells with or without XAV939 for 14 days. 4‐PBA was supplemented in drinking water. Tumor volume in each group was measured. Scale bar: 1 cm. (M) Representative photographs of livers in hMet model at the time point of 14 weeks. Yellow arrows point at visible tumors. Each liver/body weight was calculated and presented in the bar chart. (N) H&E staining of representative liver section in hMet model. The images in the black squares were enlarged with the scale bar of 200 μm. (O) Epcam distribution in liver samples of hmet model was detected by IHC. (P) Wnt5b, Fzd5, and β‐catenin expression in serial section of liver in hMetS45Y‐β‐catenin model were detected by IHC.

Various pathways are reported to be involved in CSC initiation in tumors. To identify functional pathways involved, we conducted PCR array to screen mouse stem cell relevant signaling in livers treated with or without 4‐PBA for 6 weeks in DEN plus CCl_4_ model, including 90 genes such as *Fzd1‐9, Smad1‐9 etc*. Our result suggested that nine genes were significantly overexpressed in 4‐PBA treated livers (Figure 4A). Among them, *Fzd5*, a mediator in non‐canonical β‐catenin pathway, was the most notably upregulated gene with a fold change of 8.66, indicating that Wnt‐β‐catenin signaling might be responsible for 4‐PBA‐induced CSC initiation (Figure 4A). The activation of Fzd5 was further confirmed by RT‐PCR and western blot (Figures 4B and 4C).

Next, we investigated the molecules involved in activating this pathway. As histone H3 trimethylated at lysine 4 (H3K4me3) is associated with active chromatin and gene expression, we used H3K4me3 antibody as the binding protein in chromatin immunoprecipitation sequencing (CHIP‐seq) to detect possible active gene transcription in primary hepatocyte separated from the above model. The result showed 366 peaks of 289 varied genes. Of the 366 peaks, 43 were upregulated, and 323 were downregulated (Data not shown). Gene distribution heatmap and KEGG pathway classification of the differential genes are presented (Figures 4D and 4E). We next screened 159 Wnt signaling‐related genes among 289 varied genes with peaks, of which *Wnt5b* was detected as the only overlapped gene (Figures 4F and 4G), indicating that *Wnt5b* might be the target gene of 4‐PBA. Wnt5b is a paralog of Wnt5a, serves as the ligand for Fzd5, which could be secreted to instigate the invasion of epithelial cancer cells.^36^ Interestingly, in serial liver sections of DEN model, we could detect elevated level of Wnt5b, Fzd5, and β‐catenin located along the ductular area, especially in 4‐PBA supplemented mice (Figure 4H), demonstrating that Wnt5b‐Fzd5‐β‐catenin pathway was activated by 4‐PBA.

We further confirmed the regulation of Wnt5b by 4‐PBA *in vitro*. MHCC‐LM3 was cultured in serum‐free medium supplemented with different doses of 4‐PBA (0, 1, 5, 10 mM) for 24 h. The mRNA level of *Wnt5b* elevated with the increased concentration of 4‐PBA (Figure 4I). Moreover, 4‐PBA could induce a dose‐dependent increase of secreted Wnt5b in supernatant and intracellular Wnt5b in cell lysate as detected by ELISA (Figure 4I). We also confirmed the result in Huh7 cell line under the same culture condition (Figure 4J). To further validate if Wnt5b‐β‐catenin pathway is responsible for 4‐PBA promoted LCSCs initiation and HCC development, XAV939, an inhibitor to block the activation of β‐catenin signaling was utilized. Huh7 was cultured in medium with 4‐PBA and different doses of XAV939 (0, 5, 10 μM) for 7 days. It turned out that XAV939 notably shrunk the number of spheres formed, indicating weakened stemness (Figure 4K). XAV939 also alleviated tumor burdens in Huh7 xenograft model at the presence of 4‐PBA (Figure 4L). Therefore, 4‐PBA could induce the expression of wnt5b and subsequently activate β‐catenin pathway.

In previous hMetS45Y‐β‐catenin model, enhanced hMet expression and β‐catenin activation could successfully model HCC. However, mice injected with hMet or β‐catenin mutant alone did not show any morbidity even after 22 weeks.^30^ To further investigate whether 4‐PBA exerts its function via activating β‐catenin pathway, we replaced S45Y‐β‐catenin with 4‐PBA (only pT3‐EF5a‐hMet‐V5 and pCMV/SB transposase are injected, referred to as hMet model) to see if it could mimic the tumorigenesis effect of hMetS45Y‐β‐catenin model. In hMet model, no tumors could be observed in control group or 4‐PBA‐treated group after 8 weeks. However, when the observation was prolonged to 14 weeks, multiple tumor nodules occurred in 4‐PBA treated group (Figure 4M), while no tumor was observed in control group. Liver/body ratio in 4‐PBA‐treated group was about twice that of the control group (Figure 4M). Histology of 4‐PBA‐treated liver at 14 weeks was comparable to hMetS45Y‐ β‐catenin model at 8 week (Figure 4N), which suggested that 4‐PBA could replace S45Y‐ β‐catenin to cause liver tumorigenesis. The distribution of Epcam positive liver cells was similar to hMetS45Y‐β‐catenin model (Figure 4O), which showed an intensive accumulation of Wnt5b, Fzd5, and  β‐catenin in liver samples of 4‐PBA‐treated group (Figure 4P). Taken together, we concluded that 4‐PBA activated Wnt5b‐Fzd5‐β‐catenin signaling, leading to CSCs initiation.

### 3.5 PPAR‐α signaling was involved in 4‐PBA induced tumorigenesis

**FIGURE 5:**
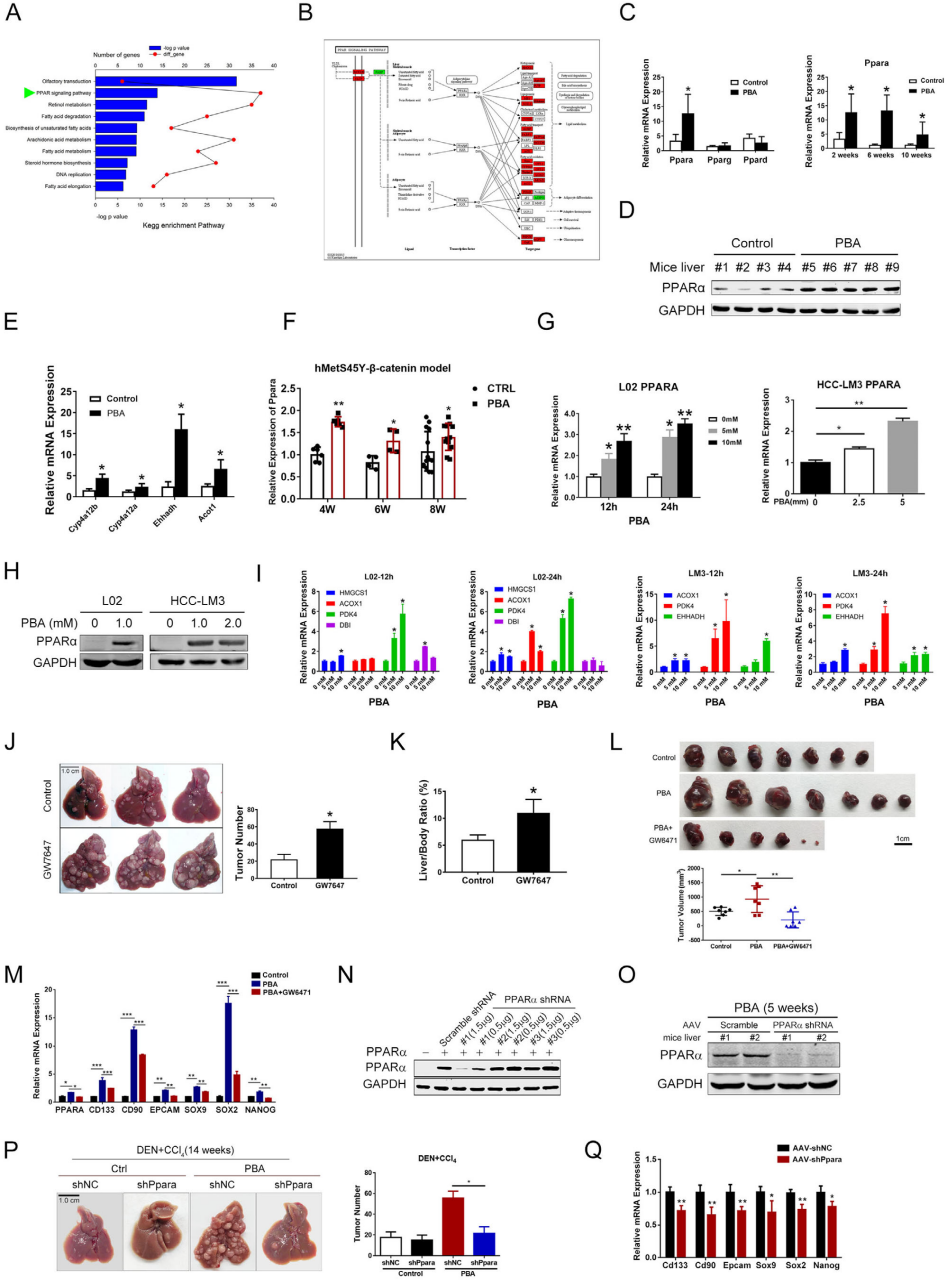
PPAR‐α signaling was involved in 4‐PBA induced tumorigenesis. (A) KEGG pathways were enriched in liver samples of DEN plus CCl_4_ model after 6 weeks with or without PBA treatment. The arrow points at PPAR signaling pathway. (B) Liver samples in DEN plus CCl_4_‐induced HCC mice model were analyzed at the transcriptional level by RNA‐seq. Thirty‐seven genes are differentially expressed in peroxisome proliferator‐activated receptors (PPARs) signaling pathway. (C) Relative expression of *Ppara, Pparg, and Ppard* in DEN+CCl_4_ mice liver was determined (left). Relative expression of PPAR‐α in DEN plus CCl_4_ mouse liver after 2 weeks, 6 weeks, and 10 weeks with or without PBA treatment was determined by RT‐PCR (right). *p < 0.05. (D) PPAR‐α protein level in liver samples was detected by western blot. (E) RT‐PCR analysis has revealed an increased level of *Cyp4a12b, Cyp4a12a, Ehhadh*, and *Acot1* in PBA treated group. (F) Relative expression of PPAR‐α in hMetS45Y‐β‐catenin model at the indicated time. (G) Cultured hepatocyte (L02) and HCC cell line MHCC‐LM3 were treated with 4‐PBA or vehicle. RT‐PCR revealed that 4‐PBAinduced overexpression of PPAR‐α. (H) The expression of PPAR‐α was detected by western blot. (I) The mRNA level of PPAR‐αrelated genes was determined by RT‐PCR. (J) Representative images of livers in control group and in mice treated with GW7647 in DEN plus CCl_4_ models for 16 weeks. Tumor number of each group is presented. *p < 0.05, **p < 0.01. (K) Liver/body ratio of J. (L) Representative images of tumors formed after Huh7 cells injection for 2 weeks in nude mice. Tumor number of each group was counted and presented. * *p* < 0.05, **p < 0.01. (M) Relative expression of CSC‐related genes (*CD133/CD90/EPCAM/SOX9/SOX2/NANOG*) in tumors of Huh7 xenograft model was determined by RT–PCR. *p < 0.05, **p < 0.01, ***p < 0.001. (N) ShRNA targeting PPAR‐α was screened. The expression of PPAR‐α was determined by western blot. (O) Western blot showed that PPAR‐α could be downregulated *in vivo* by AAV‐delivered shRNA. (P) Representative photos of livers in PBA‐free group and PBA‐treated group with AAV‐shNC or AAV‐shPPARA are presented. Tumor number in each group is presented in bar chart. *p < 0.05. (Q) Relative expression of CSC‐related genes in mice model intervened with AAV‐shPPAR‐α or AAV‐shNC was determined by RT‐PCR. *p < 0.05, **p < 0.01

To disclose the molecular mechanism underling 4‐PBA‐induced cancer stem cell initiation, liver samples in DEN plus CCl_4_ model were analyzed at the transcription level by RNA‐seq. The expression of 1,893 genes was altered under 4‐PBA treatment for 6 weeks. The pathways most highly enriched included the olfactory transduction, PPAR signaling pathway and retinol metabolism (Figure 5A). Thirty‐seven genes were differentially expressed in PPARs signaling pathway (Figure 5B), which was reported to be involved in tumorigenesis among various tissues.^37^ PPARs are ligand‐activated transcription factors of nuclear hormone receptor superfamily, comprising of PPAR‐α, PPAR‐γ, and PPAR‐β/δ. Among them, expression of PPAR‐α was specifically upregulated by 4‐PBA (Figure 5C), which was further validated by western blot (Figure 5D). To further verify the activation of PPAR‐α, we confirmed the expression of its downstream target genes in liver samples. RT‐PCR revealed an increased level of enzymes involved in fatty acid β‐oxidation such as *Ehhadh* and *Acox1* in 4‐PBA‐treated group (Figure 5E). Furthermore, in hMetS45Y‐β‐catenin model, the expression of PPAR‐α in 4‐PBA treated group was also elevated at the indicated time points, even when tumors were already formed at the 8^th^ week (Figure 5F).

Next, we evaluated the effect of 4‐PBA on the expression of PPAR‐α *in vitro*. Cultured hepatocyte (L02) and HCC cell line MHCC‐LM3 were treated with 4‐PBA or vehicle. RT‐PCR revealed that 4‐PBA induced an increase of PPAR‐α and its related genes in a time and dose‐dependent manner (Figures 5G‐5I). Notably, low concentration of 4‐PBA was able to induce significant accumulation of PPAR‐α (Figure 5H).

We further validated the activation of PPAR‐α and its role in tumorigenesis by using GW7647, a highly potent PPAR‐α selective agonist, to replace 4‐PBA in DEN induced HCC model. It turned out that GW7647 could simulate similar effect of 4‐PBA (Figure 5J), with more and larger tumor formed in livers and enhanced liver/body ratio (Figure 5K). We next explored whether PPAR‐α inhibition could reverse the tumor promoting effect of 4‐PBA. GW6471, a PPAR‐α antagonist was used to inhibit PPAR‐α activation in Huh7 xenograft model. With the administration of GW6471 for 2 weeks, the tumor promoting effect of 4‐PBA was notably impaired (Figure 5L). Analysis of the tumors suggested that GW6471 impeded CSCs expansion as indicated by lowered level of CSCs markers (Figure 5M). Furthermore, we have constructed AAV expressing shRNA targeting PPAR‐α (Figure 5N). Western blot confirmed that PPAR‐α could be downregulated *in vivo* by AAV‐delivered shRNA (Figure 5O). As expected, a single injection of AAV‐sh*Ppara* 2 weeks after DEN injection could ameliorate 4‐PBA enhanced tumorigenesis in liver, with significantly reduced tumor burden after 14 weeks (Figure 5P). The expression of CSCs‐related genes after AAV‐sh*Ppara* injection for 6 weeks in 4‐PBA group was significantly decreased (Figure 5Q). Besides, ductular reaction marked by Sox9 and CK19 was notably ameliorated both in 4‐PBA‐treated group of DDC‐diet and DEN‐induced HCC model when the expression of PPAR‐α was interfered (Figures S4A and S4B).

Our data also showed that PPAR‐α was associated with cancer stem cell initiation *in vitro*. GW6471 treatment reversed the increase of CD133 positive cells induced by 4‐PBA (Figure S4C). Meanwhile, sphere formation ability induced by 4‐PBA was impaired with GW6471, while enhanced by PPAR‐α agonist GW7647 (Figures S4D and S4E). Therefore, these data demonstrated that PPAR‐α was responsible for 4‐PBA induced CSCs initiation and tumorigenesis.

### 3.6 PPAR‐α was associated with Wnt5b‐Fzd5‐β‐catenin signaling

**FIGURE 6:**
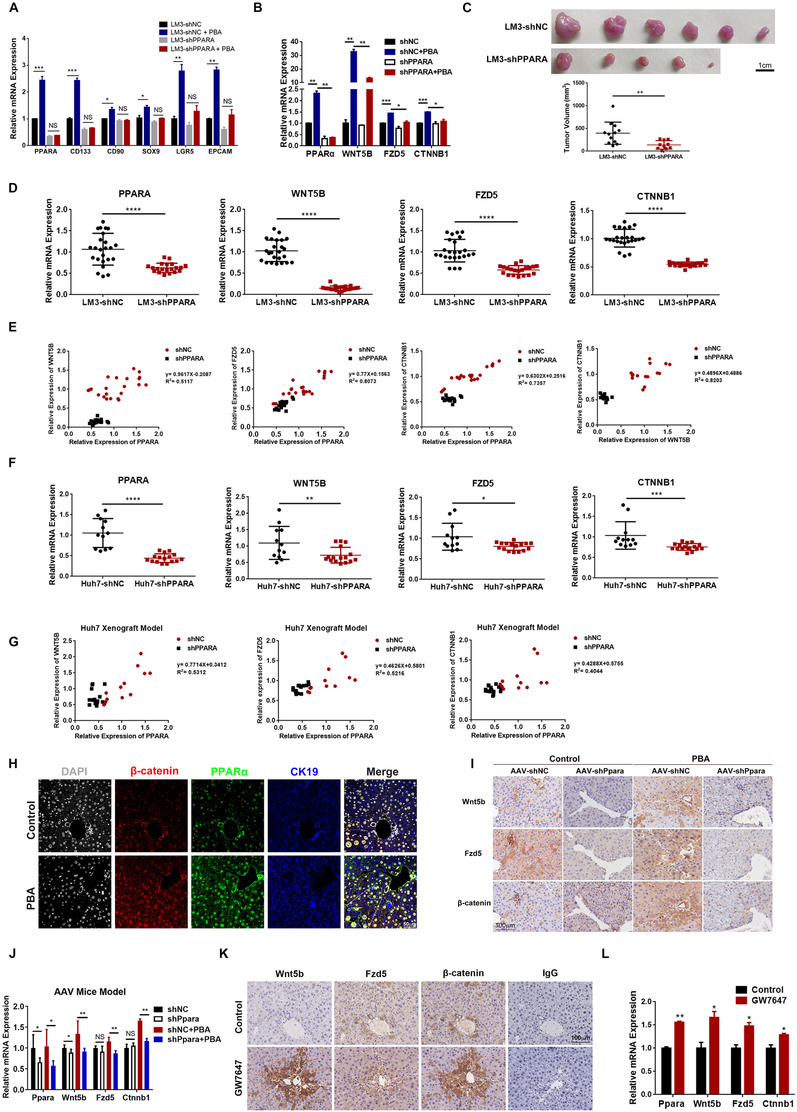
PPAR‐α was associated with Wnt5b‐β‐catenin signaling activation. (A) Relative expression of CSC‐related genes (*CD133/CD90/EPCAM/SOX9/LGR5*) in LM3‐shNC and LM3‐sh*PPARA* was determined by RT–PCR. *p < 0.05, **p < 0.01, ***p < 0.001. (B) Relative expression of *WNT5B, FZD5*, and *CTNNB1* in LM3‐shNC and LM3‐sh*PPARA* with or without 4‐PBA in culture medium for 24 h was determined by RT–PCR. (C) Representative photographs of tumors formed in Xenograft model implanted with LM3‐shNC and LM3‐sh*PPARA* with 4‐PBA supplemented in drinking water for 4 weeks. Tumor volume of each animal investigated was measured. **p < 0.01. (D) Relative expression of *PPARA, WNT5B, FZD5*, and *CTNNB1* in tumors was determined by RT‐PCR. ****p < 0.0001. (E) The relative expression of PPAR‐α in tumors formed in A was positively correlated to the expression of WNT5B, FZD5 and CTNNB1, respectively. WNT5B expression is also positively correlated with CTNNB1. (F) Relative expression of *PPARA, WNT5B, FZD5*, and *CTNNB1* in tumors formed with Huh7‐shNC, and Huh7‐sh*PPARA* was determined by RT‐PCR. (G) The relative expression of *PPARA* in tumors was positively correlated to the expression of *WNT5B, FZD5*, and *CTNNB1*, respectively. (H) Representative images of multiplexed immunohistochemistry staining of CK19 (blue), β‐catenin (red) and PPAR‐α (green) in liver section from DEN model for 6 weeks. Scale bar: 50 μm. (I) IHC staining of Wnt5b, Fzd5 and β‐catenin in serial section of liver samples from DEN model with AAV‐shNC or AAV‐sh*Ppara* were presented. Scale bar: 100 μm. (J) Relative expression of *Ppara, Wnt5b, Fzd5*, and *Ctnnb1* in liver samples in I was determined by RT‐PCR. (K) IHC staining of Wnt5b, Fzd5 and β‐catenin in serial section of liver samples from DEN model with GW7647 treatment instead of 4‐PBA. Scale bar: 100 μm. (L) Relative expression of *Ppara, Wnt5b, Fzd5*, and *Ctnnb1* in K. *p < 0.05, **p < 0.01

The above evidence has already proved that Wnt5b‐Fzd5‐β‐catenin signaling and PPAR‐α overexpression are related to 4‐PBA induced CSCs initiation. Next, we explored whether these two pathways are correlated. Firstly, PPAR‐α was knocked down in MHCC‐LM3 cell line with Lenti‐virus system (negative control: LM3‐shNC and LM3‐sh*PPARA*). Knockdown of PPAR‐α disabled 4‐PBA‐induced transcription of CSC‐related genes compared with its counterpart (Figure 6A). Next, we examined the expression of *PPARA, WNT5B, FZD5*, and *CTNNB1* in these cell lines with 4‐PBA in culture medium for 24 h. 4‐PBA has led to significantly increased transcription of *PPARA* and *WNT5B* in LM3‐shNC, as we have demonstrated previously. However, the effect of 4‐PBA was not as comparable in LM3‐sh*PPARA* as its counterpart (Figure 6B). Then, these two cell lines were injected subcutaneous in nude mice to construct xenograft model with 4‐PBA supplemented in drinking water. Mice were sacrificed 4 weeks later. Tumors formed in LM3‐sh*PPARA* injected group were less and smaller than control group (Figure 6C). With the interference of *PPARA*, mRNA levels of *WNT5B, FZD5*, and *CTNNB1* were respectively lowered, especially *WNT5B* (Figure 6D). Moreover, *PPARA* expression is positively correlated with the expression of *WNT5B, FZD5*, and *CTNNB1* relatively (Figure 6E). We also verified that *WNT5B* expression was also correlated with *CTNNB1* (Figure 6E). These results were further confirmed in Huh7‐shNC and Huh7‐sh*PPARA* cell lines, which exhibited a similar gene expression pattern and correlation between *PPARA* and *WNT5B, FZD5*, or *CTNNB1*, respectively (Figures 6F and 6G).

We further examined the activation of Wnt5b‐Fzd5‐β‐catenin pathway in liver section of DEN model. By using multiplexed immunohistochemistry to mark different proteins in one section, the images have shown more intensive staining of PPAR‐α, β‐catenin, and CK19 in 4‐PBA‐treated group (Figure 6H). In DEN model with AAV‐shPpara injection, decreased Wnt5b, Fzd5, and β‐catenin positive cells were detected in both control and 4‐PBA‐treated group in serial sections of liver samples (Figure 6I). However, the change in control group was not as notable as 4‐PBA treated group (Figure 6J). Whereas, in DEN model with PPAR‐α agonist GW7647 in place of 4‐PBA, the level of Wnt5b, Fzd5, and β‐catenin increased accordingly with a slight elevation of *Ppara* (Figures 6K and 6L). Therefore, these data strongly suggested that PPAR‐α is correlated with the activation of Wnt5b‐Fzd5‐β‐catenin signaling in the presence of 4‐PBA.

### 3.7 4‐PBA directly binded to PPAR‐α and enhanced its stabilization

**FIGURE 7:**
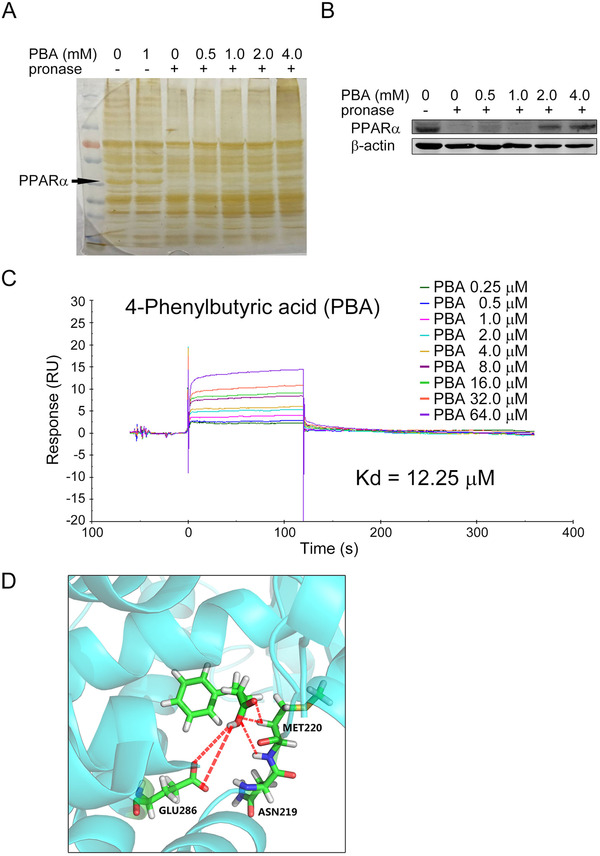
4‐PBA directly binded to PPAR‐α and enhanced its stabilization. (A) Silver‐staining showed a strong band at ∼55 kDa (as the arrow indicated) in the proteolysed extracts of 4‐PBA‐treated cells, independent of 4‐PBA concentration (0.5 mM, 1.0 mM, 2.0 mM, and 4.0 mM). (B) The band at ∼55 kDa in A was verified as PPAR‐α protein by western blotting. (C) Surface plasmon resonance analysis was used to detect the binding affinity of 4‐PBA to PPAR‐α, which revealed a concentration‐dependent manner with a dissociation constant (KD) value of 12.25 μM. (D) The computer simulation model of the direct binding of 4‐PBA to PPAR‐α. The carboxyl groups form a hydrogen bond with MET220, ASN219, and GLU286 by hydrogen bonding as a donor. The phenyl ring extends into the functional region in α‐helix.

Our data revealed that 4‐PBA could induce significant accumulation of PPAR‐α at low concentration *in vitro* and *in vivo*, thus we investigated the underlying mechanism. Drug affinity responsive target stability (DARTS) assay was performed as a means to validate binding of compounds to proteins of interest, by detecting the increase in resistance to proteolysis upon the binding of a molecule.^29^, ^38^, ^39^ Therefore, we used DARTS to explore the protection of PPAR‐α by interaction with 4‐PBA against proteolysis. Silver‐staining showed a strong band at ∼55 kDa in the proteolysed extracts of 4‐PBA‐treated cells (Figure 7A), which was further verified by western blot with PPAR‐α specific antibody (Figure 7B). The result was also confirmed by surface plasmon resonance analysis. As shown in Figure 7C, 4‐PBA could bind to the recombinant PPAR‐α protein in a concentration‐dependent manner with a dissociation constant (*K*_D_) value of 12.25 μM. 4‐PBA was docked into the active site of PPAR‐α crystal model (PDB code: 1K7L) (data not shown). In the computer simulation model presented in Figure 7D, the carboxyl groups form a hydrogen bond with MET220, ASN219, and GLU286 by hydrogen bonding as a donor. The phenyl ring extends into the functional region in α‐helix. The binding affinity was predicted to be around −4.27 kcal/moL, suggesting a modest binding potency. Therefore, 4‐PBA could not only regulate PPAR‐α transcriptionally, but also directly bind to it and enhance its stability.

### 3.8 PPAR‐α expression correlates with the prognosis of HCC patients

**FIGURE 8:**
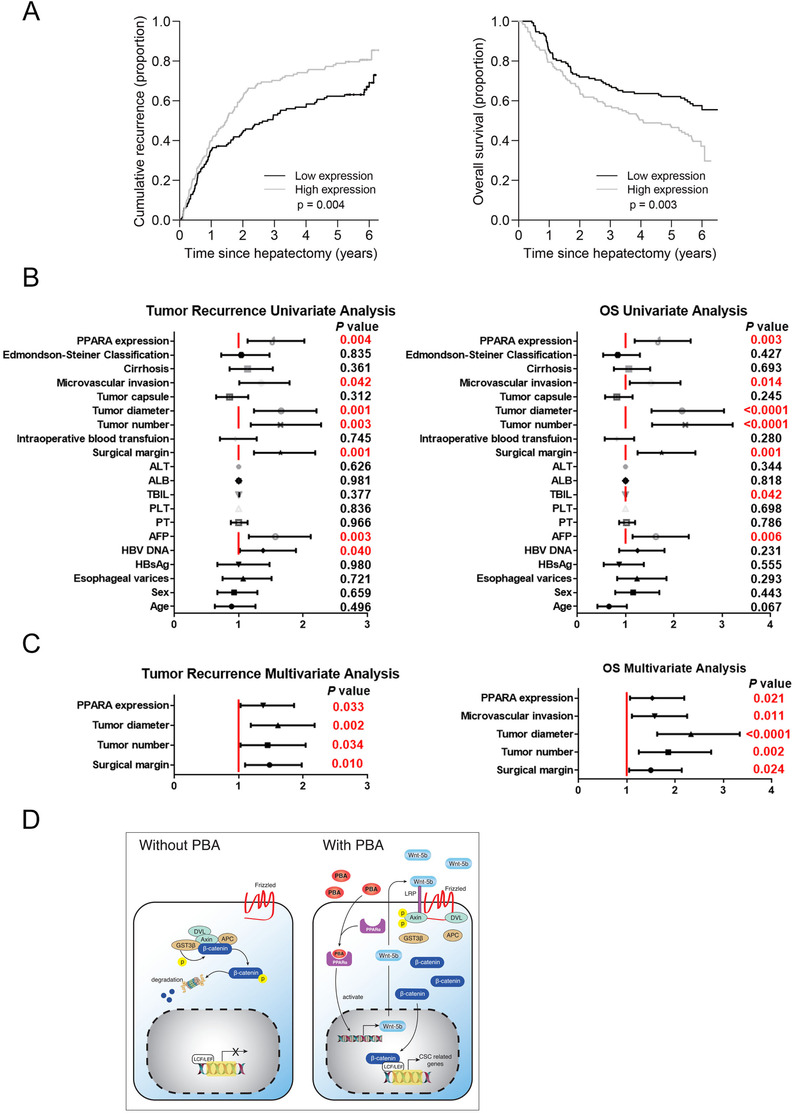
High PPAR‐α expression in HCC was associated with poor prognosis. (A) Rates of disease recurrence and overall survival in 263 HCC patients with high PPAR‐α expression (*n* = 131) and low expression (*n* = 132) were analyzed from a 6‐year follow‐up. High PPAR‐α expression correlates with worse survival in HCC patients. (B) Univariate analysis of tumor recurrence and OS. (C) Multivariate analysis of tumor recurrence and OS. (D) Schematic depiction of the mechanisms underlying 4‐PBA promoted CSCs initiation via upregulating PPAR‐α and activating Wnt5b‐Fzd5‐‐β‐catenin signaling pathway.

PPAR‐α expression of 263 HCC patient samples was evaluated by IHC staining and divided into two groups according to the median of staining intensity. Patients with higher level of PPAR‐α expression (*n* = 131) exhibited significantly increased recurrence rate and shorter overall survival (OS) compared to those with lower expression of PPAR‐α expression (*n* = 132) (Figure 8A). Clinical pathological characteristics of HCC patients were presented in Table S1. Univariate and multivariate analysis also revealed that PPAR‐α expression was an independent prognostic factor for recurrence and OS, besides tumor diameter and tumor number *etc*. (Figures 8B and 8C).

In conclusion, 4‐PBA is responsible for the accumulation of PPAR‐α both in mRNA level and protein level, activating Wnt5b‐β‐catenin pathway and initiating LCSCs, which subsequently promotes liver tumorigenesis under HCC‐inducing environment (Figure 8D).

## 4 DISCUSSION

4‐PBA, a pharmacologically available derivative of butyrate, can act at almost the same way as the unmodified BA, which is reported to have a beneficial role in prevention of malignancies. The addition of phenyl group has enriched 4‐PBA with chaperon‐like properties, concerning with restoration of tissue homeostasis in multiple researches. According to the success of preclinical studies, PBA has been approved by the U.S. FDA for clinical use in urea‐cycle disorders as an ammonia scavenger. It has also been tested in clinical trials for the treatment of other diseases such as thalassemia and cystic fibrosis.^40^, ^41^, ^42^ In recent studies, 4‐PBA has been reported to reduce hepatocellular lipid accumulation and hepatotoxicity during liver injury *in vitro*.^28^, ^43^ To investigate the role of 4‐PBA on liver, we studied its long‐time effect under HCC‐inducing environment. Unexpectedly, our data revealed that 4‐PBA could promote HCC initiation in chemical‐induced mice model via inflammation and ER stress independent mechanism. We have also observed that 4‐PBA cast a protective effect during DEN‐induced liver injury demonstrated by alleviated inflammation and fibrosis. Nevertheless, 4‐PBA increased liver tumorigenesis through initiation of LCSCs. Significant ductal reaction could be observed as soon as 2 weeks after 4‐PBA administration. Meanwhile, the expression of CSC markers was notably increased in 4‐PBA treated liver. Thus, the protective role of 4‐PBA via reducing ER stress during liver fibrosis is not as comparable as its role on LCSCs initiation in HCC models.

In short‐term driver gene‐induced HCC model (referred to as hMetS45Y‐β‐catenin), hMet, and mutant β‐catenin concomitant expression could lead to liver tumor development in mice.^30^ 4‐PBA administration obviously accelerated liver tumor onset with earlier microscopic nodules (4W) and notable macroscopic tumors after 8 weeks. When S45Y‐β‐catenin was spared from this model, no visible liver tumor was observed as long as 22 weeks of plasmids injection according to the previous report.^30^ However, when 4‐PBA was supplemented, massive liver tumor could be observed in every animal that investigated (eight of eight) after 14 weeks of plasmids injection. Although the onset of liver tumor was prolonged, and the intra‐tumoral hemorrhage was not as severe as the original model, 4‐PBA could indeed serve as a replacement of mutant β‐catenin in promoting tumorigenesis, which could be considered as indirect evidence that it could activate β‐catenin pathway. Therefore, we could conclude that 4‐PBA also played pivotal role in tumorigenesis in this short‐term HCC model.

In our study, we have observed significant increase of PPAR‐α induced by 4‐PBA both in mRNA level and protein level *in vitro* and *in vivo*. According to previous studies, 4‐PBA is a non‐classical peroxisome proliferator which could induce pleiotropic effects including transcriptional activation through histone deacetylase enzymes (HDAC) inhibition.^23^ This could be a possible explanation for the upregulation of PPAR‐α in mRNA level. Besides, by DARTS assay, we also found that 4‐PBA could directly bind to PPAR‐α and prevent it from proteolysis, which provided a novel insight for PPAR‐α regulation.

PPAR‐α plays a key role in maintaining glucose and lipid homeostasis as well as in cell proliferation, differentiation, and inflammatory responses.^44^ The decrease of PPAR‐α is widely implicated in preclinical models of non‐alcoholic steatohepatitis (NASH), NAFLD, and alcoholic liver disease.^45^, ^46^, ^47^, ^48^ Therefore, in many studies, PPAR‐α is considered as a potential therapeutic target for these metabolism‐related liver diseases.^48^, ^49^, ^50^ Activation of PPAR‐α was considered to ameliorate ethanol‐induced steatohepatitis and liver fibrosis in mice^51^, ^52^, ^53^ and exhibited a protective role in NAFLD or NASH models.^54^, ^55^ However, the role of PPAR‐α in hepatocarcinogenesis remained controversial. PPAR‐α agonists caused increased incidence of liver tumors via PPAR‐α‐mediated manner as indicated by the resistance of *Ppara*‐null mice to liver cancer under the stimulation of PPAR‐α ligand.^56^ Whereas, PPAR‐α humanized mice showed resistance to liver cancer tumorigenesis, probably due to different response to PPAR‐α ligand (Wy‐14,643) in different species.^57^, ^58^ Although whether PPAR‐α could directly cause HCC remained unclear, a bunch of  studies have linked PPAR‐α activation as part of the mechanism for HCC development.^59^, ^60^, ^61^ For example, Keratin 23 was considered a PPAR‐α dependent, MYC‐amplified oncogene that might remove rate‐limiting constraints on hepatocyte proliferation and lead to liver cancer.^60^ Previous study has also reported a synergized effect of PPAR‐α and glucocorticoid receptor to promote erythroid progenitor self‐renewal.^62^ In this study, by using high throughput method to screen the pathways involved in 4‐PBA‐treated samples, we have identified PPAR‐α as the regulator of downstream genes. PCR array revealed *Fzd5* as one of the most altered genes in 4‐PBA group (Figure S5A). CHIP‐seq also suggested *Wnt5b* as the only candidate of Wnt signaling pathway‐related genes with H3K4me3 modification under 4‐PBA treatment. *In vitro* study validated that 4‐PBA induced the release of Wnt5b in supernatant and its accumulation in cytoplasm in a dose‐dependent manner. Further study revealed a positive correlation of PPAR‐α expression and the activation of Wnt5b signaling pathway. However, if 4‐PBA could regulate Wnt5b transcription via ways other than PPAR‐α needs to be further investigated.

Although 4‐PBA alone could not initiate the onset of liver tumor, it has a tumor‐promoting role under HCC‐inducing background. Since the role of Wnt signaling pathway is well understood in liver cancer, it is worth noticing that the connection between PPAR‐α and Wnt signaling activation might limit the use of therapeutic modulators such as PPAR‐α agonist in certain patients. Meanwhile, PPAR‐α expression predicts poor prognosis in HCC patients, suggesting that more concern should be taken regarding the use of 4‐PBA in clinics due to its role in promoting PPAR‐α expression. Nevertheless, therapeutic modulators such as PPAR‐α antagonist might serve as a potential adjuvant drug for patients under long‐term 4‐PBA treatment.

## CONFLICT OF INTEREST

The authors have no conflict of interest to declare.

## AUTHOR CONTRIBUTIONS

Conception and design: Le‐Xing Yu, Wen Wen, and Hong‐Yang Wang. Development of methodology: Xiao‐Fei Chen, Shu‐Zhen Chen, and Yan Ling. Acquisition of data (provided animals, provided facilities, etc.): Shu‐Zhen Chen, Yan Ling, Yu‐Ting Song, and Jiao‐Jiao Tang. Collection of patients sample and clinical data: Can Chen. Patients’ Follow‐up and data sorting: Ya‐Ping Dong. Analysis and interpretation of data (e.g., statistical analysis, biostatistics, computational analysis): Shu‐Zhen Chen, Qi‐Qi Cao, Han Yu, and Yu‐Shan Miao. Writing, review, and/or revision of the manuscript: Shu‐Zhen Chen and Hong‐Yang Wang. Administrative, technical, or material support (i.e., reporting or organizing data, constructing databases): Zhe‐Cai Fan, Jun‐Yan Tao, and Satdarshan P.S. Monga. Study supervision: Wen Wen and Hong‐Yang Wang.

## Supporting information

Supporting Information

## Data Availability

The data that support the findings of this study are available from the corresponding author upon reasonable request.
