# Supplementary material for: 4‐phenylbutyric acid promotes hepatocellular carcinoma via initiating cancer stem cells through activation of PPAR‐α
Source: Clin Transl Med. 2021 May 1;11(4):e379. doi: 10.1002/ctm2.379 (PMC8087947; doi:10.1002/ctm2.379)
Supplement: Supplementary file 1 — Supporting Information [file CTM2-11-e379-s001.docx]

**4-Phenylbutyric Acid Promotes Hepatocellular Carcinoma Via Cancer Stem Cells Initiation Through Activation of Peroxisome Proliferator-Activated Receptor-α**

Shu-Zhen Chen^1,2^‡, Yan Ling ^1,2^‡, Le-Xing Yu^1,2^‡, Yu-Ting Song^1,2,6^‡, Xiao-Fei Chen^4^, Qi-Qi Cao^1,2^, Han Yu^1,2^, Can Chen^1,2,3^, Jiao-Jiao Tang^7^, Zhe-Cai Fan^1^, Yu-Shan Miao^1,2^, Ya-Ping Dong^1,2,3^, Jun-Yan Tao^8^, Satdarshan P.S. Monga^8,9^, Wen Wen^1,2^*, Hong-Yang Wang^1-3,5-7^*

^1^National Center for Liver Cancer, Second Military Medical University, Shanghai, China

^2^International Cooperation Laboratory on Signal Transduction, Eastern Hepatobiliary Surgery Hospital, Second Military Medical University, Shanghai, China

^3^Fujian Medical University, Fuzhou, Fujian Province, China

^4^School of Pharmacy, Second Military Medical University, Shanghai, China

^5^State Key Laboratory of Oncogenes and Related Genes, Shanghai Cancer Institute, Renji Hospital, School of Medicine, Shanghai Jiaotong University, Shanghai, China

^6^Model Animal Research Center, Nanjing University, Nanjing, Jiangsu Province, China

^7^ Cancer Research Center, The First Affiliated Hospital of USTC, University of Science and Technology of China, Hefei, Anhui, China

^8^ Department of Pathology, University of Pittsburgh, Pittsburgh, PA

^9^ Department of Medicine, University of Pittsburgh, Pittsburgh, PA

‡These authors contributed equally to this work

***Correspondence:** Dr. Hongyang Wang ([hywangk@vip.sina.com](mailto:hywangk@vip.sina.com)), International Cooperation Laboratory on Signal Transduction, Eastern Hepatobiliary Surgery Hospital, 225 Changhai Road, Shanghai 200438, China. Tel: 86-21-81875361. Fax number: 86-21-65566851.

**Dr. Wen Wen (**[wenwen_smmu@163.com](mailto:wenwen_smmu@163.com)), International Cooperation Laboratory on Signal Transduction, Eastern Hepatobiliary Surgery Hospital, 225 Changhai Road, Shanghai 200438, China. Tel: 86-21-81875363. Fax number: 86-21-65566851.

**Table of contents**

Materials and Methods 3

Supporting Tables 9

Supporting Table 1 Clinical pathological characteristics of HCC patients 9

Supporting Table 2 Univariable analysis of tumor recurrence and OS 11

Supporting Table 3 Univariable analysis of tumor recurrence and OS 12

Supporting Table 4 Sequence of primers for Real-time PCR 13

Supporting Table 5 Antibody list 14

Supplementary Figures 15

References 24

## Materials and Methods

**Chemicals and Regents**

4-Phenylbutyric acid，GW7647, GW6471 were purchased from Sigma-Aldrich (St. Louis, MO, USA). SimpleChIP® Plus Sonication Chromatin IP Kit was purchased from Cell Signaling Inc. (Beverly, MA, USA). RT² Profiler PCR Array for Mouse Stem Cell Signaling was purchased from QIAGEN. Adeno-associated virus (AAV) expressing short hairpin RNA (shRNA) targeting PPAR-α was perchased from OBiO (Shanghai).

For the driver gene induced HCC model, all the constructs used for mouse injection were kindly provided by Dr. Satdarshan Monga and Dr. Junyan Tao from University of Pittsburgh, including pT3-EF5a-hMet-V5 and pCMV/sleeping beauty transposase (SB) (1). Human β-catenin clone with S45Y mutation described previously (2) were cloned into pT3-EF5α plasmid via the Gateway PCR cloning strategy (Invitrogen, Carlsbad, CA) along with an addition of a Myc tag as indicated previously (3). The plasmids were hydrodynamic injected as described previously (4). All the plasmids used for *in vivo* experiments were purified using the Endotoxin free Maxi prep kit (Sigma-Aldrich, St.Louis, MO) before being injected into the mice.

**Real-time RT (reverse transcription) PCR**
Total mRNAs were extracted from cells and tissues using TRIzol reagent (Invitrogen).The concentration and quality of RNAs were determined by Nanodrop 2000 (Thermo Fisher Scientific). mRNAs reversely transcribed to cDNAs with PrimeScript RT Master Mix (Takara, Japan) according to the manufacturer’s instructions. Quantitative PCR was performed using SYBR Green PCR Kit (Applied Biosystems, Foster City, CA). β-actin was used as an internal control. The real-time PCR was carried out with SYBR Green Master Mix (Takara, Japan) in the StepOnePlusTM Real-time PCR system (Applied Biosystems, USA). PCR amplification of genes was performed under the conditions: denature at 95℃ for 30 s, anneal at 60℃ for 60 s, and extended at 95℃ for 5s; reactions were carried out for 45 cycles. For each sample, reactions were duplicated and the average mRNA level of each gene was determined using the 22DDCt method. The primers used for RT-PCR are listed in Supporting Table 2.

**Western blot analysis**

Samples of cell or lysates were separated by 8–12% SDS-PAGE gels and transferred onto polyvinyldifluoride membranes. After blocked with 5% nonfat milk and 0.1% Tween 20 in TBS for 1 h, the membrane was then incubated at 4°C for overnight with the primary antibodies. The primary antibody anti-PPAR alpha (1:200) were purchased from Santa Cruz, USA; the anti-b-actin antibody (1:200) were obtained from Santa Cruz Biotechnology, Inc. Immunocomplexes were incubated with the fluorescein-conjugated secondary antibody, and then detected using Odyssey fluorescence scanner (Li-Cor, Lincoln, NE). All the antibodies used in this study are listed in Supporting Table 3.

**Cell culture**

Cell lines Huh7, MHCC-LM3, HepG2 and L02 were obtained from Shanghai Cell Bank (Shanghai, China) and routinely cultured in Dulbecco’s modified Eagle’s medium (Invitrogen, Carlsbad, CA) supplemented with 10% fetal bovine serum (Gibco, Waltham, MA) within a humidified incubator containing 5% CO2 at 37℃.

**Immunohistochemistry and Immunofluorescence Staining**

Mice tissues were divided and fixed in phosphate-buffered neutral formalin, embedded in paraffin, and cut into 5μm-thick sections. Briefly, the sections were incubated with primary antibody at 4℃ overnight and then with horseradish peroxidase–conjugated secondary antibody at 37℃ for 30 minutes. The sections were finally incubated with diaminobenzidine and counterstained with hematoxylin for detection. Assessment of the staining was based on the percentage of positively stained cells and the staining intensity using software Image-Pro Plus 6.0 (Media Cybernetics, Inc.). Frozen sections of fresh mice liver tissue were incubated with rabbit anti-CD133 followed by fluorescent staining with Fluor 555-conjugated antirabbit IgG (Invitrogen)，and 40,6- diamidino-2-phenylindole (DAPI) was applied to show the nucleus. Representative images were captured with an Olympus IX70.

**Chromatin immunoprecipitation sequencing (ChIP-seq) assays**

The H3k4me3-ChIPed-DNA was purified by a QIAquick PCR Purification Kit (QIAGEN) as instructed. 30ng of DNA was used for CHIP-Seq. For ChIP-seq library preparation, enriched DNA samples were performed with end-pair of the DNA, adding ‘‘A’’ bases to the DNA, linking sequencing adapters to DNA, adaptor linked DNA amplification and gel purification for ChIP-seq by a NEB Next Ultra DNA Library Prep Kit. Purified DNA was sequenced on the Illumina HiSeq 4000 platform.

ChIP-Seq reads were preprocessed using Trimmomatic (5), mapped to the mouse genome mm10 using Bowtie2 (6), uniquely mapped reads located both upstream and downstream of transcription start statistical using deeptools (7), and enriched using MACS2 (8). The protein-binding motif was identified using MEME-ChIP software (9). Difference peaks analysis using Manorm software (10) with default parameters p-value < 0.05 and |log2 (M-value) | > 1. ChIP-Seq service was provided by OE Biotech Co. (Shanghai, China).

**Drug Affinity Responsive Target Stability (DARTS)**

We use a range of pronase concentrations (1:100, 1:300, 1:1000, 1:3000, and 1:10,000 ratios) to determine the optimal amount needed to partially digest the interested PPAR-α protein. Based on our observation, 1:300 is determined for the protein of interest. 4-PBA solutions were added directly to the protein lysates in different concentration from 0.5mM to 4mM, incubated at room temperature for 1 hour. Corresponding pronase stock solution was added into an aliquot of each protein samples in 1 min intervals. After incubating in 37℃ water bath in 30 min, 4×SDS was added into each sample to stop the digestion, heated immediately then placed on ice. The fractions were analyzed by silver staining and traditional SDS-PAGE as described before.

**Surface plasmon resonance analysis**

Purified human PPAR-α (50μg/mL) was injected onto the HisCap Sensor Chip for immobilization. Various concentrations of 4-PBA in running buffer (1X PBS, 0.5 % DMSO) were passed over the chip to produce response signals. The association and dissociation rate constants were calculated using the GE Biacore T2000 Evaluation software. The ratio of the association and dissociation rate constants was determined as the binding affinity.

**Computational modeling**

The structure of the target protein PPAR-α (PDB code: 1K7L) was obtained from Protein Data Bank ([www.pdb.org](http://www.pdb.org)). Lib-dock in Discovery Studio 3.0 was used to conduct molecular docking between active components and target proteins. Crystal water was removed, and hydrogen atoms were added according to the protonation state of chemical groups at pH 7.0. The co-crystallized molecules binding with target proteins were regarded as positive drug whose dock scores were defined as cutoff value. If the score of a component was higher than the cutoff value, this component can be considered binging well with the relevant protein.

# Flow cytometry assay.

# Huh7 cells (2 × 10^5^) were cultured in each well of six-well plates at 70% to 80% confluence. Following 4-PBA treatment for 24 h, and then harvested and suspended in PBS containing 1% BSA and were stained with anti-CD133-PE or anti-CD133-APC (Meltenyi, Germany) antibodies for 30 min on ice. Flow cytometry analysis was performed using Moflo-XDP (Beckman Coulter, Fullerton, CA) equipped with Summit 5.1 Software.

# Immobilization of PPAR-α on sensor surface and SPR screening

# Purified protein PPAR-α was purchased from Qiangyao biological company (China). SPR analysis was carried out on a Biacore T200 system (GE Healthcare, Sweden). PPAR-α were diluted in 10 mM sodium acetate (pH 4.0) to final concentration of 100ug/ml and immobilized by the amine coupling method on a CM5 sensor chip according to the manufacturer’s protocol. The target immobilization level of ligand was 19616.8 RU. The specificity for PPAR-α binding was characterized by GW6471 which was used as positive control. The running buffer is phosphate buffer saline (PBS). Samples were then injected to the PPAR-α sensor surface for 60 s at a flow rate of 30 μL/min. Sensorgrams of PBA were recorded and analyzed.

| Supporting TablesSupporting Table 1 Clinical pathological characteristics of HCC patients | | | | | | |
| --- | --- | --- | --- | --- | --- | --- |
|  |  |  | **PPARA** | |  |  |
| **Variables** | **Total** |  | **Low** | **High** |  | **P-value** |
| **Age,years** | | | | | | |
| <=60 | 206 (78.3%) |  | 99 (75.0%) | 107 (81.7%) |  | 0.23 |
| >60 | 57 (21.7%) |  | 33 (25.0%) | 24 (18.3%) |  |  |
| **Sex** | | | | | | |
| Female | 66 (25.1%) |  | 38 (28.8%) | 28 (21.4%) |  | 0.20 |
| Male | 197 (74.9%) |  | 94 (71.2%) | 103 (78.6%) |  |  |
| **Esophageal varices** | | | | | | |
| Absent | 210 (79.8%) |  | 103 (78.0%) | 107 (81.7%) |  | 0.54 |
| Present | 53 (20.2%) |  | 29 (22.0%) | 24 (18.3%) |  |  |
| **HBsAg** | | | | | | |
| Negative | 41 (15.6%) |  | 18 (13.6%) | 23 (17.6%) |  | 0.40 |
| Positive | 222 (84.4%) |  | 114 (86.4%) | 108 (82.4%) |  |  |
| **Log HBV DNA** | | | | | | |
| <=4 | 192 (73.0%) |  | 96 (72.7%) | 96 (73.3%) |  | 1.0 |
| >4 | 71 (27.0%) |  | 36 (27.3%) | 35 (26.7%) |  |  |
| **AFP, g/L** | | | | | | |
| <=400 | 183 (69.6%) |  | 97 (73.5%) | 86 (65.6%) |  | 0.18 |
| >400 | 80 (30.4%) |  | 35 (26.5%) | 45 (34.4%) |  |  |
| **PT, seconds** | | | | | | |
| Median (IQR) | 12.1 (11.4 - 12.8) |  | 12.1 (11.4 - 12.9) | 12.1 (11.4 - 12.6) |  | 0.32 |
| **PLT, *10^9^/L** | | | | | | |
| Median (IQR) | 146.0 (102.5 - 185.0) |  | 155.0 (97.0 - 192.2) | 144.0 (105.5 - 177.0) |  | 0.093 |
| **TBIL,mol/L** | | | | | | |
| Median (IQR) | 13.5 (10.5 - 18.0) |  | 13.6 (10.3 - 17.6) | 13.5 (10.5 - 18.0) |  | 0.23 |
| **ALB, g/L** | | | | | | |
| Median (IQR) | 41.7 (39.2 - 44.0) |  | 41.5 (39.0 - 43.8) | 42.1 (39.5 - 44.0) |  | 0.46 |
| **ALT, U/L** | | | | | | |
| Median (IQR) | 37.0 (26.2 - 58.6) |  | 39.3 (26.3 - 63.7) | 34.9 (25.4 - 50.5) |  | 0.25 |
| **Surgical margin, cm** | | | | | | |
| <1 | 103 (39.2%) |  | 53 (40.2%) | 50 (38.2%) |  | 0.80 |
| >=1 | 160 (60.8%) |  | 79 (59.8%) | 81 (61.8%) |  |  |
| **Transfusion** | | | | | | |
| No | 169 (64.3%) |  | 85 (64.4%) | 84 (64.1%) |  | 1.0 |
| Yes | 94 (35.7%) |  | 47 (35.6%) | 47 (35.9%) |  |  |
| **Tumor number** | | | | | | |
| Single | 204 (77.6%) |  | 111 (84.1%) | 93 (71.0%) |  | 0.012 |
| Multiple | 59 (22.4%) |  | 21 (15.9%) | 38 (29.0%) |  |  |
| **Tumor diameter, cm** | | | | | | |
| <=5 | 173 (65.8%) |  | 96 (72.7%) | 77 (58.8%) |  | 0.020 |
| >5 | 90 (34.2%) |  | 36 (27.3%) | 54 (41.2%) |  |  |
| **Tumor capsule** | | | | | | |
| Incomplete | 140 (53.2%) |  | 62 (47.0%) | 78 (59.5%) |  | 0.048 |
| Complete | 123 (46.8%) |  | 70 (53.0%) | 53 (40.5%) |  |  |
| **Microvascular invasion** | | | | | | |
| Absent | 154 (58.6%) |  | 81 (61.4%) | 73 (55.7%) |  | 0.38 |
| Present | 109 (41.4%) |  | 51 (38.6%) | 58 (44.3%) |  |  |
| **Cirrhosis** | | | | | | |
| No | 112 (42.6%) |  | 58 (43.9%) | 54 (41.2%) |  | 0.71 |
| Yes | 151 (57.4%) |  | 74 (56.1%) | 77 (58.8%) |  |  |
| **Tumor differentiation** | | | | | | |
| I-II | 212 (80.6%) |  | 104 (78.8%) | 108 (82.4%) |  | 0.53 |
| III-IV | 51 (19.4%) |  | 28 (21.2%) | 23 (17.6%) |  |  |
|  | | | | | | |

**Abbreviations:** HR, hazard ratio; CI, confidence interval; HBeAg, hepatitis B e antigen; AFP, alpha-fetoprotein; TBIL, total bilirubin; ALT, alanine aminotransferase. PVTT, portal vein tumor thrombus

**Supporting Table 2 Univariable analysis of tumor recurrence and OS**

| **Variable** | **Tumor recurrence** | | | |  | **OS** | | | |
| --- | --- | --- | --- | --- | --- | --- | --- | --- | --- |
|  | **P** | **HR** | **95.0% CI** | |  | **P** | **HR** | **95.0% CI** | |
| **Age,**years  ≤60/>60 | 0.496 | 0.89 | 0.63 | 1.26 |  | 0.067 | 0.66 | 0.42 | 1.03 |
| **Sex**  male/female | 0.659 | 0.93 | 0.67 | 1.29 |  | 0.443 | 1.16 | 0.79 | 1.70 |
| **Esophageal varices**  presence/absence | 0.721 | 1.07 | 0.75 | 1.51 |  | 0.293 | 1.24 | 0.83 | 1.85 |
| **HBsAg**  positive/negative | 0.980 | 1.00 | 0.67 | 1.48 |  | 0.555 | 0.87 | 0.55 | 1.38 |
| **HBV DNA**, log10, IU/mL  ≤4/>4 | 0.040 | 1.38 | 1.02 | 1.89 |  | 0.231 | 1.25 | 0.87 | 1.81 |
| **AFP**, ug/l  ≤20/>20 | 0.003 | 1.57 | 1.16 | 2.12 |  | 0.006 | 1.63 | 1.15 | 2.31 |
| **PT, seconds** | 0.966 | 1.00 | 0.88 | 1.14 |  | 0.786 | 1.02 | 0.87 | 1.20 |
| **PLT, 10^9^/L** | 0.836 | 1.00 | 1.00 | 1.00 |  | 0.698 | 1.00 | 1.00 | 1.00 |
| **TBIL,**μmol/l | 0.377 | 1.00 | 1.00 | 1.01 |  | 0.042 | 1.00 | 1.00 | 1.01 |
| **ALB, g/L** | 0.981 | 1.00 | 0.98 | 1.03 |  | 0.818 | 1.00 | 0.98 | 1.03 |
| **ALT,**u/l | 0.626 | 1.00 | 1.00 | 1.00 |  | 0.344 | 1.00 | 1.00 | 1.00 |
| **Surgical margin,cm**  <1/≥1 | 0.001 | 1.65 | 1.24 | 2.19 |  | 0.001 | 1.75 | 1.25 | 2.45 |
| **Intraoperative blood**  **transfusion**  yes/no | 0.745 | 0.95 | 0.71 | 1.28 |  | 0.280 | 0.82 | 0.57 | 1.18 |
| **Tumor number**  single/multiple | 0.003 | 1.65 | 1.19 | 2.28 |  | <.0001 | 2.24 | 1.55 | 3.22 |
| **Tumor diameter,**cm  ≤5/>5 | 0.001 | 1.66 | 1.24 | 2.21 |  | <.0001 | 2.17 | 1.54 | 3.04 |
| **Tumor capsule**  complete/incomplete | 0.312 | 0.86 | 0.65 | 1.15 |  | 0.245 | 0.82 | 0.58 | 1.15 |
| **Microvascular invasion**  presence/absence | 0.042 | 1.35 | 1.01 | 1.79 |  | 0.014 | 1.53 | 1.09 | 2.14 |
| **Cirrhosis**  presence/absence | 0.361 | 1.14 | 0.86 | 1.53 |  | 0.693 | 1.07 | 0.76 | 1.51 |
| **Edmondson-Steiner Classification**  I-II/III-IV | 0.835 | 1.04 | 0.73 | 1.48 |  | 0.427 | 0.84 | 0.54 | 1.30 |
| **PPARA expression**  high/low | 0.004 | 1.52 | 1.14 | 2.02 |  | 0.003 | 1.67 | 1.19 | 2.35 |

**Abbreviations:** OS, overall survival; HR, hazard ratio; CI, confidence interval; HBeAg, hepatitis B e antigen; AFP, alpha-fetoprotein; TBIL, total bilirubin; ALT, alanine aminotransferase. PVTT, portal vein tumor thrombus

**Supporting Table 3 Multivariate analysis of tumor recurrence and OS**

| **Variable*** | **Tumor recurrence** | | | |  | **OS** | | | |
| --- | --- | --- | --- | --- | --- | --- | --- | --- | --- |
|  | **P** | **HR (95.0% CI)** | | |  | **P** | **HR (95.0% CI)** | | |
| **Surgical margin,cm，**<1/≥1 | 0.010 | 1.48 | 1.10 | 1.98 |  | 0.024 | 1.50 | 1.05 | 2.14 |
| **Tumor number,** single/multiple | 0.034 | 1.45 | 1.03 | 2.04 |  | 0.002 | 1.86 | 1.25 | 2.75 |
| **Tumor diameter**, cm, ≤5 vs. >5 | 0.002 | 1.61 | 1.19 | 2.18 |  | <.0001 | 2.33 | 1.63 | 3.34 |
| **Microvascular invasion** ,presence/absence | - | - | - | - |  | 0.011 | 1.58 | 1.11 | 2.25 |
| **PPARA expression**, high/low | 0.033 | 1.38 | 1.03 | 1.86 |  | 0.021 | 1.53 | 1.07 | 2.19 |

**Abbreviations:** HR, hazard ratio; CI, confidence interval. AFP, alpha-fetoprotein; PVTT, portal vein tumor thrombus

*Only significant factors in the univirate analysis were shown and subjected to multivariate analysis.

### Supporting Table 4 Sequence of primers for Real-time PCR

| **Primer** | **Forward Sequence (5' to 3')** | **Reverse Sequence** |
| --- | --- | --- |
| Mouse | | |
| *Epcam* | CTGGCGTCTAAATGCTTGGC | CCTTGTCGGTTCTTCGGACTC |
| *Cd90* | TGCTCTCAGTCTTGCAGGTG | TGGATGGAGTTATCCTTGGTGTT |
| *Bmi* | ATCCCCACTTAATGTGTGTCCT | CTTGCTGGTCTCCAAGTAACG |
| *Oct4* | AGAGGATCACCTTGGGGTACA | CGAAGCGACAGATGGTGGTC |
| *Sox2* | CGGCACAGATGCAACCGAT | CCGTTCATGTAGGTCTGCG |
| *Cd133* | CCTTGTGGTTCTTACGTTTGTTG | CGTTGACGACATTCTCAAGCTG |
| *Stat3* | CACCTTGGATTGAGAGTCAAGAC | AGGAATCGGCTATATTGCTGGT |
| *Wnt5b* | AGATAGGTAGCCGAGAGACTGC | GGTAGCCGTACTCCACGTTG |
| *Fzd5* | GGTGTGCCAGGAAATCACG | CACAAGCGGCCAGAATTGG |
| *Ctnnb1* | ATGGAGCCGGACAGAAAAGC | CTTGCCACTCAGGGAAGGA |
| *Ppara* | GATGTCACACAATGCAATTCG | CAGCAGTGGAAGAATCGGAC |
| *Pparg* | GGAAGACCACTCGCATTCCTT | GTAATCAGCAACCATTGGGTCA |
| *Ppard* | TCCATCGTCAACAAAGACGGG | ACTTGGGCTCAATGATGTCAC |
| *Actb* | GGCTGTATTCCCCTCCATCG | CCAGTTGGTAACAATGCCATGT |
| Human | | |
| *CD90* | CTAGTGGACCAGAGCCTTCG | GCACGTGCTTCTTTGTCTCA |
| *CD133* | GCCACCGCTCTAGATACTGC | TGTTGTGATGGGCTTGTCAT |
| *EPCAM* | CTGCCAAATGTTTGGTGATG | AAAGCCCATCATTGTTCTGG |
| *NANOG* | ATGCCTCACACGGAGACTG | GTTCTGGAACCAGGTCTTCAC |
| *SOX2* | TGCACAACTCGGAGATCAGC | CTGCATCATGCTGTAGCTGC |
| *SOX9* | GTGCTCAAAGGCTACGACTG | CAGCTGCTCCGTCTTGATG |
| *WNT5B* | CGCTTCGCCAAGGAGTTTG | TGCCATCTTATACACAGCCCT |
| *PPARA* | TGTCACACAACGCGATTCG | CTTGTTCTGGATGCCATTGG |
| *LGR5* | CTCCCAGGTCTGGTGTGTTG | GAGGTCTAGGTAGGAGGTGAAG |
| *FZD5* | CCGTTCGTGTGCAAGTGTC | GAAGCGTTCCATGTCGATGAG |
| *CTNNB1* | CATCTACACAGTTTGATGCTGCT | GCAGTTTTGTCAGTTCAGGGA |
| *ACTB* | CTACCTCATGAAGATCCTCACC | CACAGGACTCCATGCCCAG |

### Supporting Table 5 Antibody list

| **Antibody** | **Origin** | **Catalog number** |
| --- | --- | --- |
| HNF-4α | Cell Signaling Technology | 3113S |
| CK19 | Abcam | ab15463 |
| Ki-67 | Abcam | ab15580 |
| Sox9 | EMD Millipore Corp | AB5535 |
| CD133/1-PE | Miltenyi Biotec | 130-080-801 |
| CD133/1-APC | Miltenyi Biotec | 130-090-826 |
| CD133/2-APC | Miltenyi Biotec | 130-090-854 |
| CD24 | Biolegend | B225997 |
| Wnt5b | Santa Cruz Biotechnology | sc-376249 |
| FZD5 | Abcam | ab75234 |
| β-catenin | Cell Signaling Technology | 8480P |
| PPAR-α | Abcam | ab24509 |
| Myc-tag | Cell Signaling Technology | 2278S |
| Epcam | Abcam | ab71916 |
| GAPDH | Cell Signaling Technology | 5174S |
| α-SMA | Sigma-Aledrich | A5228 |
| F/480 | Abd serotec | ABCA0651031 |
| Hspa5 | Cell Signaling Technology | 3177S |
| p-PERK | Santa Cruz Biotechnology | sc-9477 |
| ATF4 | Cell Signaling Technology | 11815S |
| CHOP | Cell Signaling Technology | 5554S |
| p-elF2α | Cell Signaling Technology | 9721S |
| PARP | Cell Signaling Technology | 9532S |

## Supplementary Figures

#
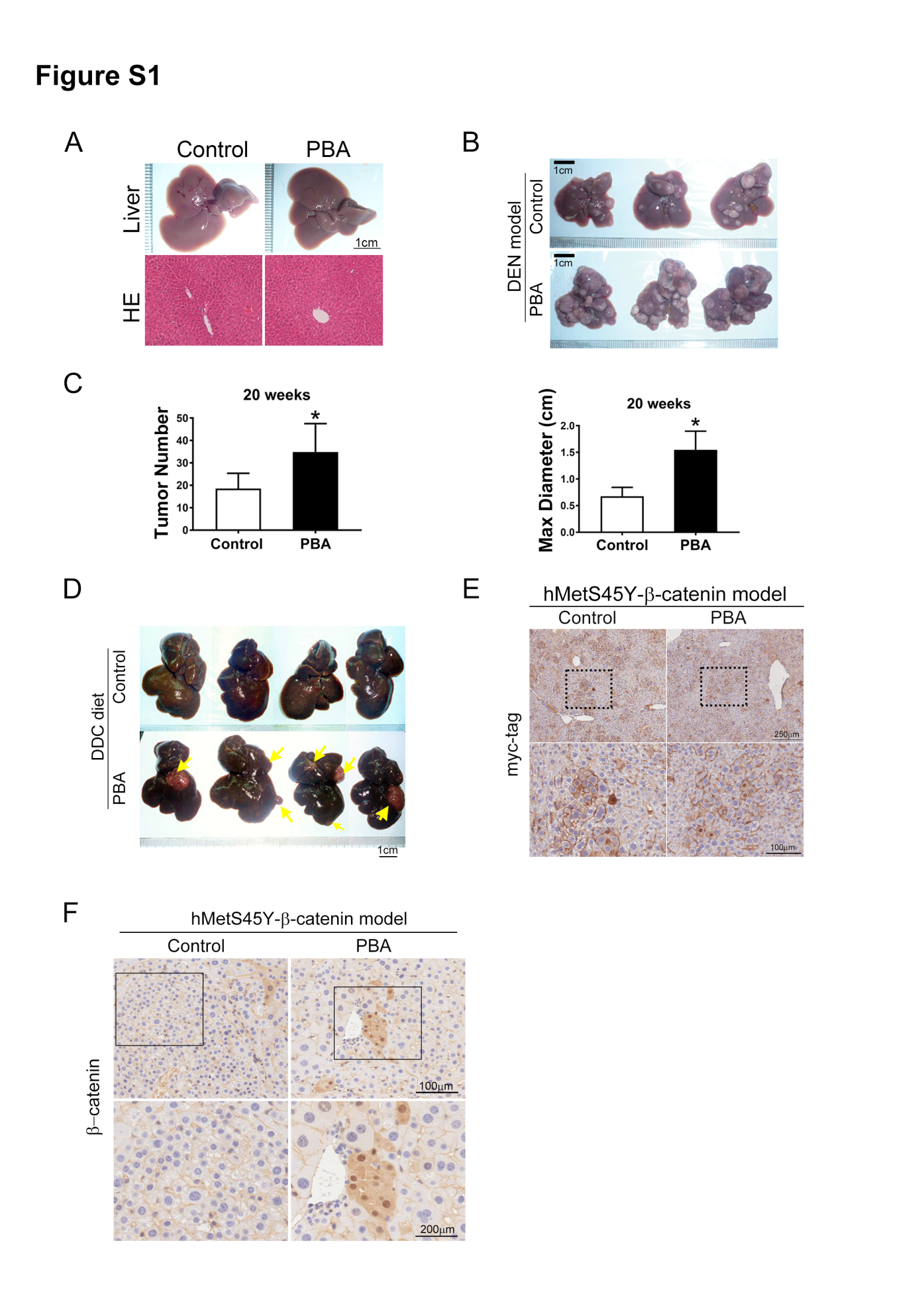


**Supplement Fig.1** **4-PBA promotes hepatocarcinogenesis in mice model.**

(A) Representative images and HE staining of livers in control group and 4-PBA single treatment group after 40 weeks. No tumor was observed in both groups. (B) Representative images of livers in DEN plus CCl_4_ model after 20 weeks in PBA treated group and the control group. Scale bar: 1cm. (C) Tumor number and max diameter of tumors in PBA treated group and the control group were measured in DEN plus CCl_4_ model at the time point indicated in A. ** P* <0.05. (D) Representative images of livers in DDC diet model after 57 weeks in PBA treated group and the control group. The yellow arrows points at tumors on livers. Scale bar: 1cm. (E) Representative images of IHC staining of myc-tag in livers of S45Y-β-catenin model. (F) Representative images of IHC staining of β-catenin in livers of S45Y-β-catenin model.

**
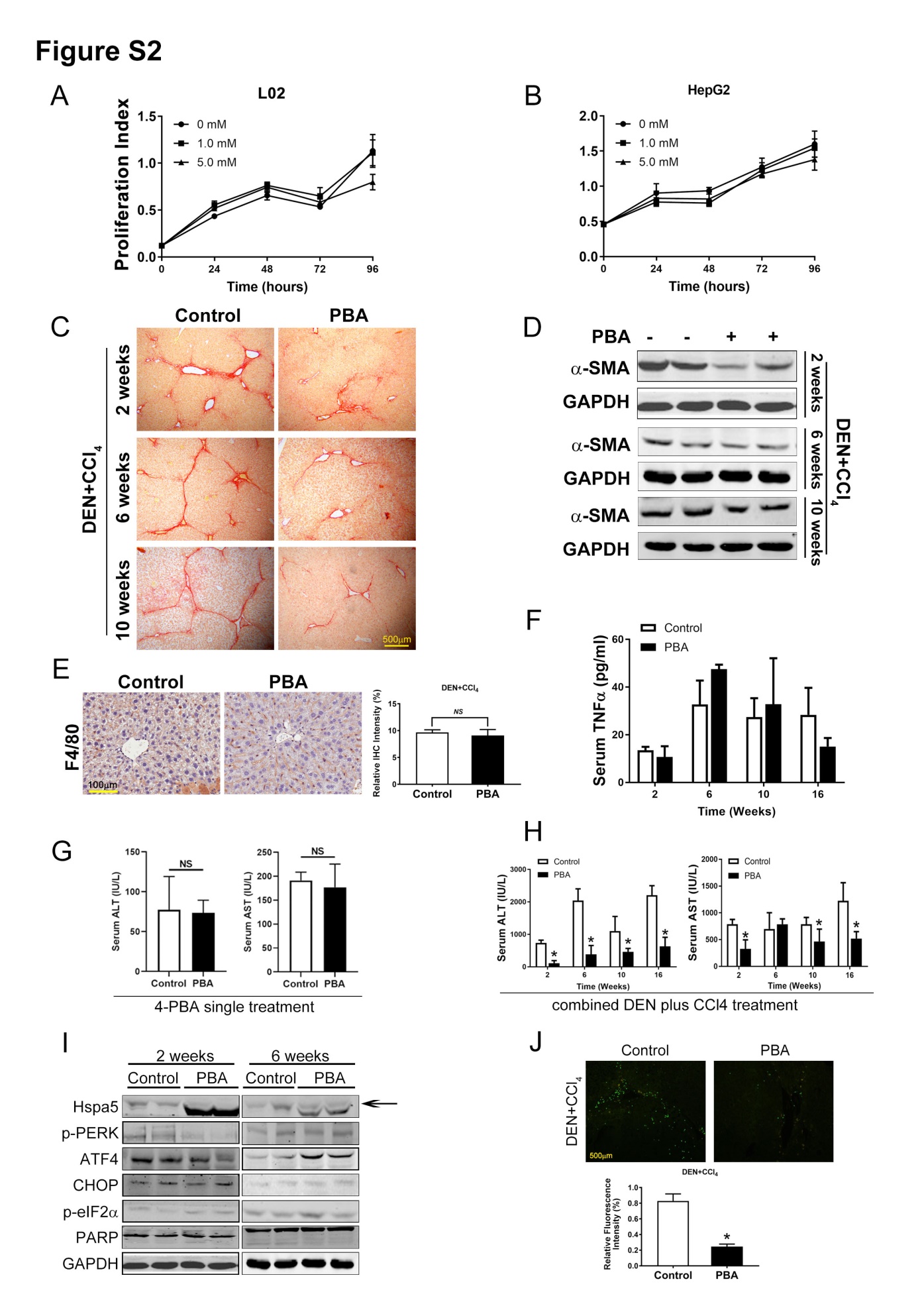
**

**Supplement Fig.2** **4-PBA promoted liver tumorigenesis via inflammation and ER stress independent way.**

(A-B) L02 and HepG2 were cultured in medium supplemented with 4-PBA. Cell proliferation was measured every 24 hours for a total of 96 hours. Proliferation index was calculated compared with cell numbers at the starting point. (C) Sirius red staining has revealed alleviated liver fibrosis in DEN plus CCl_4_ model supplemented with 4-PBA for indicated time points. Scale bar: 500μm (D) α-SMA protein level in liver samples was determined by Western Blot after 4-PBA administration at indicated time points. (E) F4/80 staining of liver sections in DEN model supplemented with 4-PBA for 6 weeks. IHC intensity was measured in 4 vision fields per liver sample. Each group has more than 4 mice livers measured. (F) The level of TNFα in serum was determined at the indicated time point after 4-PBA administration. (G) (H) The level of ALT and AST in serum was determined at the indicated time points after 4-PBA administration. (I) Protein level of Hspa5, p-PERK, ATF4, CHOP, p-elF2a and PARP was determined by Western Blot after 4-PBA administration at indicated time points. The arrow points at the band of Hspa5. (J) TUNEL assay revealed that DEN-induced apoptosis was significantly reduced in the liver of 4-PBA supplemented mice.


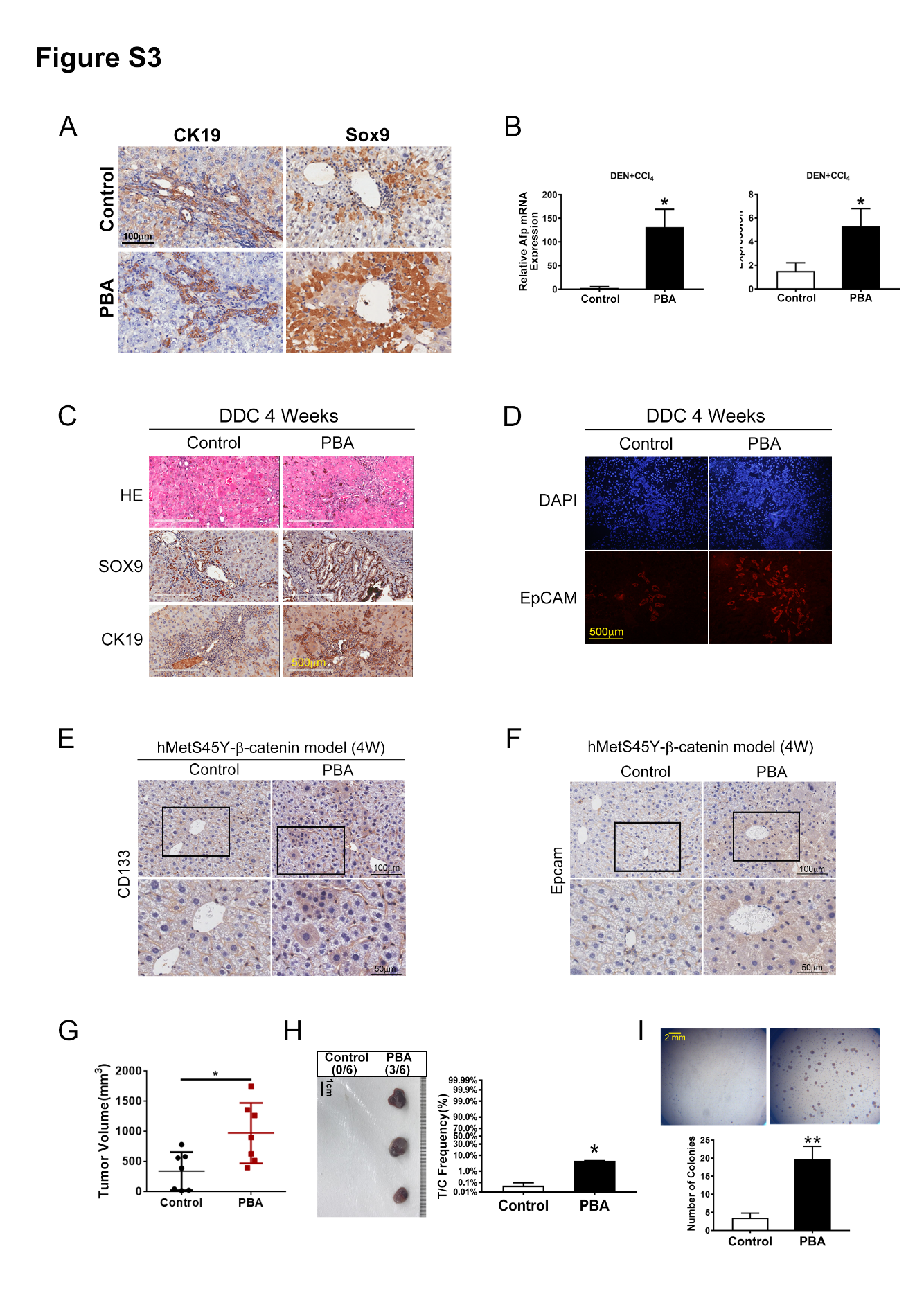


**Supplement Fig.3** **4-PBA promoted tumorigenesis via initiation of cancer stem cells.**

(A) Representative images of CK19 and Sox9 staining of the liver in DEN plus CCl_4_ HCC model after 2 weeks with or without PBA treatment. Scale bar: 100μm. (B) Relative expression of Afp and H19 in DEN model supplemented with 4-PBA for 6 weeks. (C) H&E, CK19 and SOX9 staining of liver samples in DDC-diet model with 4-PBA supplemented in drinking water for 4 weeks. Scale bar: 100μm. (D) CSCs biomarker EpCAM was detected by immunofluorescence in DDC diet model. Scale bar: 100μm. (E-F) CD133 and Epcam expression in liver samples of hMetS45Y-β-catenin model are detected by IHC. (G) Tumor volumes were measured and calculated in Figure 3D. ** P* <0.05. (H) Limiting dilution assay was performed using HepG2 in NOD-SCID mice with 100, 500, 2000, 5000, 10,000 cells implanted respectively. After 8 weeks, tumors occurred in 3 out of 6 mice in 4-PBA treated group with 10,000 cells implanted. No tumor was observed in control group. (I) More colonies of HepG2 were formed in soft agar with 4-PBA. The Number of colonies was presented in the bar chart. ** *P* <0.01.


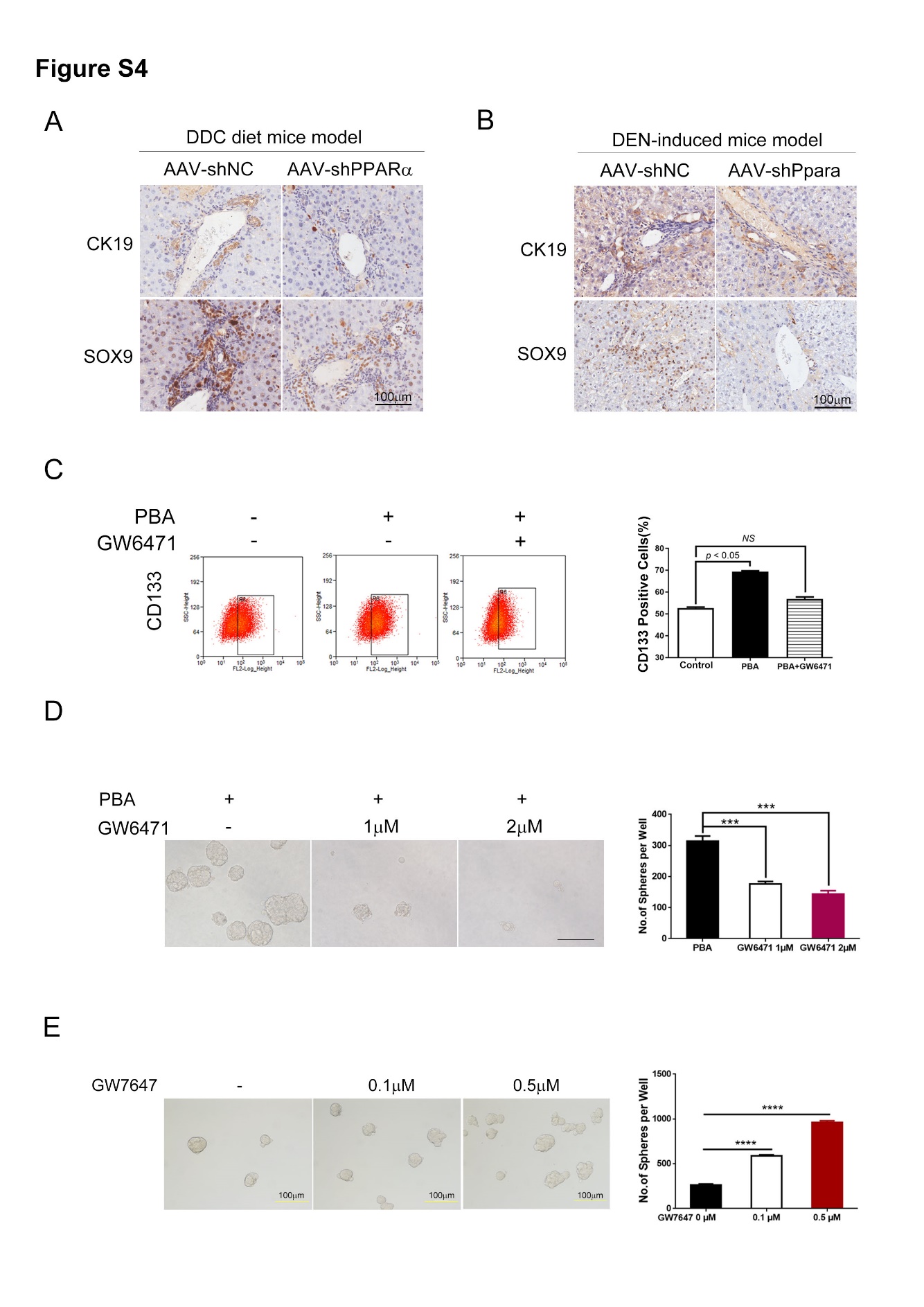


**Supplement Fig.4 Inhibition of PPAR-α reversed 4-PBA-induced activation of CSCs.**

(A) Ductular reaction markers CK19 and Sox9 staining of representative liver sections in DDC diet model treated with PBA for 2 weeks. Scale bar: 100μm. (B) Ductular reaction markers CK19 and Sox9 staining of representative liver sections in previous model treated with PBA for 2 weeks. Scale bar: 100μm. (C) CD133 positive Huh7 cells after GW6471 treatment for 24 hours were detected by flow cytometry analysis. The bar chart shows the quantification of CD133 positive cells. (D) Representative photos of spheres formed with Huh7 cells treated with different doses of GW6471 for 7 days. The bar chart shows the average amount of spheres formed per well. *** P <0.001. Scale bar: 100μm. (E) Representative photos of spheres formed with Huh7 cells treated with different doses of GW7647 for 7 days. The bar chart shows the average amount of spheres formed per well. **** P <0.0001. Scale bar: 100μm.


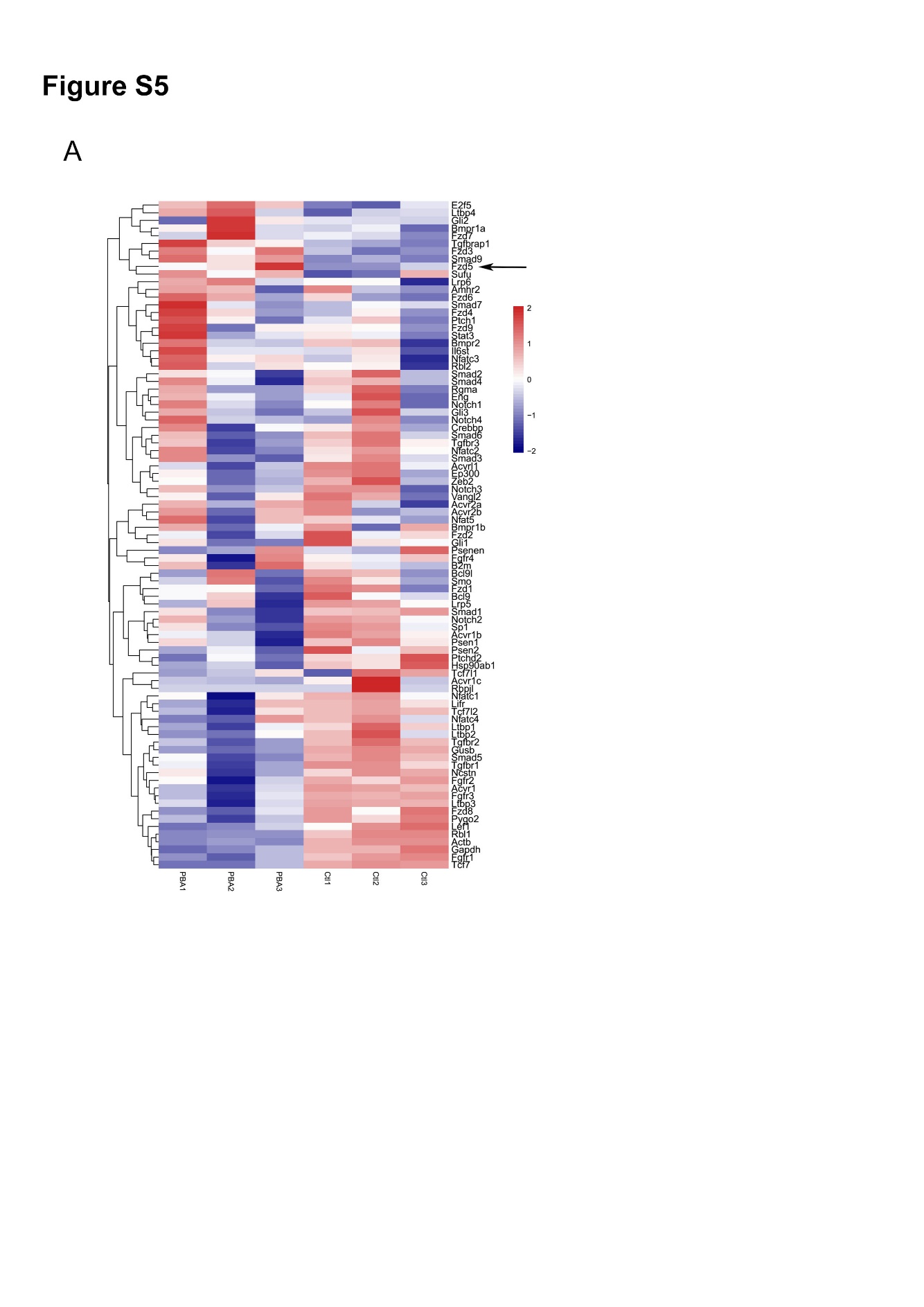


**Supplement Fig.5** Heatmap of mouse CSCs related genes in PCR array determined by RNA-seq. The black arrow points at the gene *Fzd5*.

## References

1. Patil MA, Lee SA, Macias E, Lam ET, Xu C, Jones KD, Ho C, et al. Role of cyclin D1 as a mediator of c-Met and beta-catenin-induced hepatocarcinogenesis. Cancer Res 2009;69:253-261.

2. Lee JM, Yang J, Newell P, Singh S, Parwani A, Friedman SL, Nejak-Bowen KN, et al. beta-Catenin signaling in hepatocellular cancer: Implications in inflammation, fibrosis, and proliferation. Cancer Lett 2014;343:90-97.

3. Tao J, Xu E, Zhao Y, Singh S, Li X, Couchy G, Chen X, et al. Modeling a human hepatocellular carcinoma subset in mice through coexpression of met and point-mutant beta-catenin. Hepatology 2016;64:1587-1605.

4. Tao J, Calvisi DF, Ranganathan S, Cigliano A, Zhou L, Singh S, Jiang L, et al. Activation of beta-catenin and Yap1 in human hepatoblastoma and induction of hepatocarcinogenesis in mice. Gastroenterology 2014;147:690-701.

5. Bolger A M, Lohse M, Usadel B. Trimmomatic: a flexible trimmer for Illumina sequence data[J]. Bioinformatics, 2014, 30(15):2114.

6. Langmead B, Salzberg S L. Fast gapped-read alignment with Bowtie 2[J]. Nature Methods, 2012, 9(4): 357.

1. Ramírez F, Dündar F, Diehl S, et al. deepTools: a flexible platform for exploring deep-sequencing data[J]. Nucleic Acids Research, 2014, 42(W1): W187-W191.
2. Zhang Y, Liu T, Meyer C A, et al. Model-based analysis of ChIP-Seq (MACS)[J]. Genome Biology, 2008, 9(9): R137.
3. Bailey T L, Boden M, Buske F A, et al. MEME SUITE: tools for motif discovery and searching[J]. Nucleic Acids Research, 2009, 37(suppl_2): W202-W208.
4. Shao Z, Zhang Y, Yuan G C, et al. MAnorm: a robust model for quantitative comparison of ChIP-Seq data sets[J]. Genome Biology, 2012, 13(3): R16.
